# Fermi Level Shifts of Organic Semiconductor Films in Ambient Air

**DOI:** 10.1021/acsami.4c13674

**Published:** 2025-01-08

**Authors:** Xian’e Li, Qilun Zhang, Yongzhen Chen, Xianjie Liu, Slawomir Braun, Mats Fahlman

**Affiliations:** 1 Laboratory of Organic Electronics, Department of Science and Technology (ITN), Linköping University, Norrköping SE-60174, Sweden; 2 Wallenberg Wood Science Center, Department of Science and Technology (ITN), Linköping University, Norrköping SE-60174, Sweden

**Keywords:** organic semiconductors, work function shifts, Kelvin probe, near-ambient pressure XPS, ambient air, doping

## Abstract

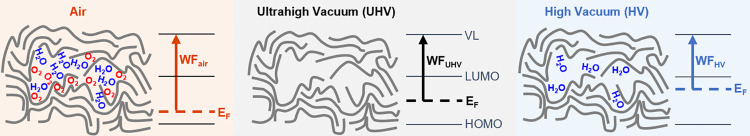

Here, the Fermi level (*E*_F_) shifts of several donor and acceptor materials in different atmospheres are systematically studied by following the work function (WF) changes with Kelvin probe measurements, ultraviolet photoelectron spectroscopy, and near-ambient pressure X-ray photoelectron spectroscopy. Reversible *E*_F_ shifts are found with the trend of higher WFs measured in ambient air and lower WFs measured in high vacuum compared to the WFs measured in ultrahigh vacuum. The *E*_F_ shifts are energy level and morphology-dependent, and two mechanisms are proposed: (1) competition between p-doping induced by O_2_ and H_2_O/O_2_ complexes and n-doping induced by H_2_O; (2) polar H_2_O molecules preferentially modifying the ionization energy of one of the frontier molecular orbitals over the other. The results provide a deep understanding of the role of the O_2_ and H_2_O molecules in organic semiconductors, guiding the way toward air-stable organic electronic devices.

## 1 Introduction

Organic semiconductors (OSs) have received considerable attention due to their great potential in scalable roll-to-roll manufacturing of various organic electronic devices.^1,2^ Despite the remarkable progress on material synthesis and device optimization in the past decades, air stability is still the Achilles’ heel of these organic electronic devices for their lab-to-fab translation.

For organic photovoltaics (OPVs), the lab-scale devices are usually fabricated in vacuum or in an inert gas-filled glovebox, while the industrial-scale ones are usually exposed to the ambient air where O_2_ and H_2_O have been identified to be the critical stress factors to affect the material and device stability.^3−5^ Intensive efforts have been dedicated to the irreversible chemical structure degradation and morphology changes of these OSs that occur upon long-term air exposure.^6−10^ In contrast, less attention has been paid to the reversible doping effect universally found at the early stage of air exposure, which is reported to be responsible for the degradation of device performance through the formation of charge traps that cause a lower charge carrier mobility and a higher charge recombination rate.^11−15^

Moreover, the complicated interactions of O_2_ and H_2_O with OS materials keep the debate on doping mechanisms unresolved. Generally, p-doping by air is more commonly observed in OSs than n-doping, and O_2_ is widely accepted as the main p-dopant.^16−20^ Some researchers also noted the key roles of H_2_O molecules in the doping process. Ho et al. find that both O_2_ and H_2_O are involved in the photoinduced doping process in thiophene-based OSs, probably via O_2_(H_2_O)_*n*_ clusters, while O_2_ or H_2_O acting alone cannot create a significant photodoping effect.^21^ Blom et al. proposed that hydrated oxygen complexes and water clusters are possible origins of electron and hole traps, respectively,^22,23^ such that a universal trap-free energy window for OSs can be established according to these trap energies. Kemerink et al. on the other hand suggested that the dielectric effect of water penetrating nanovoids in OS films has priority compared to any energy-level-dependent redox reactions; thus, trap energies generally lie ∼0.3–0.4 eV above the highest occupied molecular orbital (HOMO) and below the lowest unoccupied molecular orbital (LUMO) level of the OSs, irrespective of their energy levels.^24^ There are also some interesting discussions on the mutual passivating interactions between O_2_ and H_2_O molecules.^22,25−27^ Most studies primarily investigate the doping effects of OS films in inert atmospheres with minimal water and oxygen, conditions that significantly differ from those in ambient air. However, ambient air is typically used for large-scale printing and fabrication of devices.

This study systematically investigates Fermi level (*E*_F_) shifts of several donors and nonfullerene acceptors (NFAs) in different atmospheres, revealing p- or n-doping trends. By comparing work function (WF) values measured in ambient air or high vacuum (HV) using a Kelvin probe (KP) with those in ultrahigh vacuum (UHV) via ultraviolet photoelectron spectroscopy (UPS), we find that most OSs exhibit p-doping trends (*E*_F_ shifting toward the HOMO) in ambient air, where O_2_ and H_2_O coexist, and n-doping trends (*E*_F_ shifting toward the LUMO) in HV, primarily due to residual H_2_O in the films. The doping trends, observed by in situ WF evolution during air-vacuum cycling in the KP chamber, are nearly reversible. A slower p-doping rate is observed in OS films with deeper energy levels and more ordered morphologies. The hole-only device measurements reveal distinct doping behaviors between donors and acceptors during air exposure. Near-ambient pressure X-ray photoelectron spectroscopy (NAP-XPS) experiments show that water molecules lower the WFs of OS films and passivate the p-doping effects aroused by oxygen. Finally, we propose two scenarios—(1) competing p/n doping effects and (2) water dielectric effects—to explain the *E*_F_ shift behavior of OSs in ambient air. The general principles governing *E*_F_ shifts in OS films could guide the development of air-stable OSs and the optimization of air-processed OPV devices, paving the way for lab-to-fab translation.

## 2 Results and Discussion

### 2.1 WF Shifts of OS Films in Ambient Air, HV, and UHV

To investigate the doping effect by ambient air on OSs for high-efficiency OPV systems, several donors (P3HT, PTB7-Th, TQ1, PBDB-T, PM6, PM7, and PTO2) and NFAs (IEICO, IEICO-4F, O-IDTBR, Y1, Y6, ITIC, and IT4F) with different energy levels are selected. The chemical structures of these materials are depicted in Figure S1 in the Supporting Information. Each film, with a thickness between 20 and 100 nm, is spin-coated onto an indium tin oxide (ITO) substrate inside an N_2_-filled glovebox (O_2_ < 1 ppm, H_2_O < 1 ppm). The films are then transferred to the KP chamber in ambient air, kept in darkness. The WF of each sample is measured sequentially under ambient air (≈20 °C, relative humidity: 20–40%), HV (∼1 × 10^–5^ mbar) using KP. Finally, the samples are transferred from the KP chamber in ambient air to the UPS vacuum chamber and measured under UHV (<1 × 10^–9^ mbar). This approach enables us to examine the dependence of each OS film’s WF on various atmospheric conditions: ambient air, where both O_2_ and H_2_O coexist; HV conditions, in which residual H_2_O remains in the films^28−30^ due to its slower effusion rate compared to O_2_^31^; and UHV conditions, which are virtually free of O_2_ and H_2_O.

For reliable KP calibration, a stainless-steel reference probe with minimum contaminant adsorption and stable WF in both air and vacuum is applied in this work. Additionally, the WF of the reference probe (WF_ref_) is calibrated against several substrates with highly stable WFs, showing only minor variations (<0.1 eV) from ambient air to high vacuum, such as freshly cleaved surface of highly ordered pyrolytic graphite (HOPG), ITO, and aluminum with a native oxide layer (Al/AlO_*x*_) (Figure S2). A fitting line with a fixed slope of −1 is drawn among the data points of calibration substrates whose contact potential difference values (e·CPD) measured by KP in HV are plotted against their WF values measured by UPS (WF_UHV_) in UHV. WF_ref_ is determined to be 4.5 eV from the intercept of this fitted line (where e·CPD = 0 eV), with a standard error of 0.05 eV for the intercept value. Additionally, the 95% confidence band of the calibration line is shown as a guide to assess the reliability of WF deviations for each material measured in air or HV compared with their WF_UHV_ values. Data points outside this band indicate significant WF deviations. This approach averages individual reference deviations and enhances calibration reliability and accuracy. The WFs of each OS film in ambient air and HV conditions are resolved from the CPD values by KP (Figures S2 and S3). Furthermore, to ensure a reliable comparison, KP and UPS measurements must be conducted meticulously to further minimize systematic errors and other factors beyond atmospheric conditions. These include potential beam damage to films,^32^ UV-induced work function shifts during UPS measurements,^33,34^ and other calibration issues.^35,36^ For example, some researchers found the ITO substrate to be unstable under UV light during UPS measurements,^34^ but in our work, this instability is not observed (Supplementary Note 1, Table S1, Figure S4), likely due to the low-dose, monochromatized and defocused UV light source used in our setup. These concerns are discussed further in detail in the Supporting Information (Supplementary Notes 1–3). Considering the experimental errors for UPS (±0.05 eV), KP (±0.01 eV), and the standard error for WF_ref_ determination (±0.05 eV), the overall error bar for the WF deviation of each material is ±0.07 eV (see Supplementary Note 2). Additionally, both KP and UPS measurements are carried out in the dark to avoid the influence from light-induced charges.

**Figure 1:**
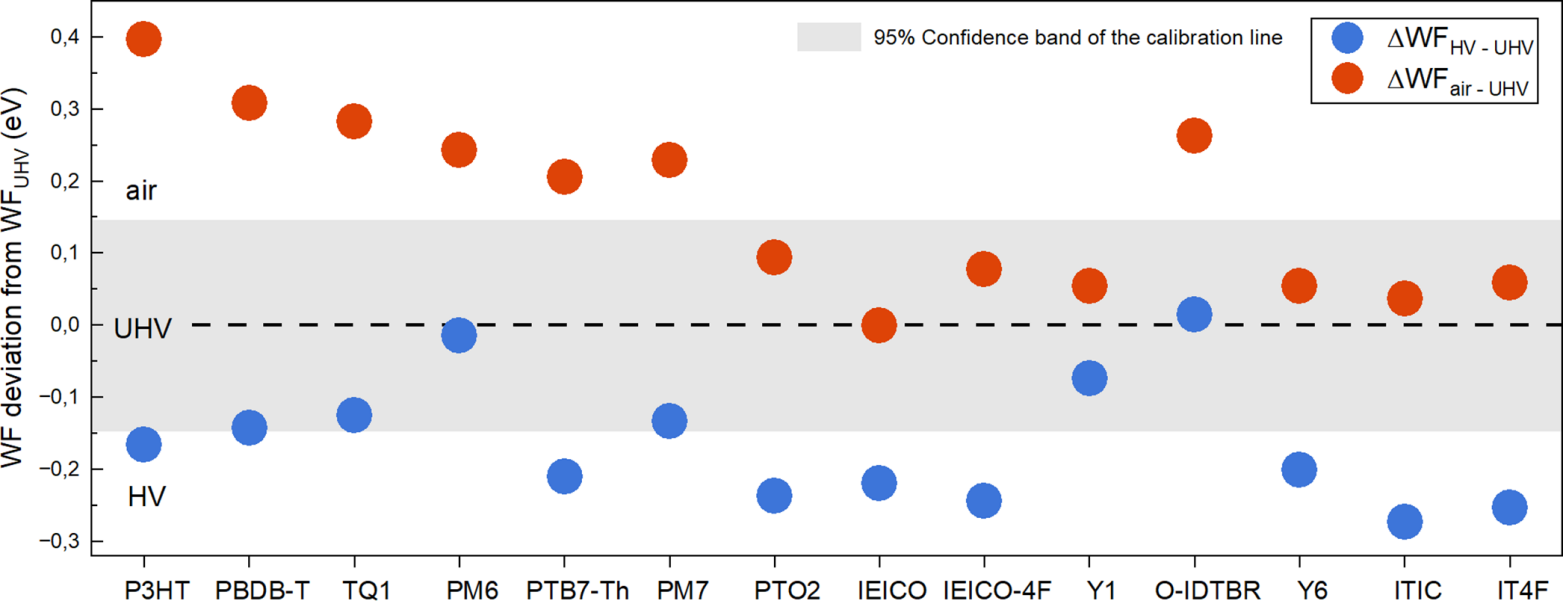
Work function (WF) deviations of samples measured in air (red dots) and high vacuum (blue dots) compared to their values in ultrahigh vacuum (UHV). The dashed calibration line and its 95% confidence band are shown for reference. The deviations are defined as ΔWF_air-UHV_ = WF_air_ – WF_UHV_ and ΔWF_HV-UHV_ = WF_HV_ – WF_UHV_, with each measurement having an error bar of ±0.07 eV. Data points outside the 95% confidence region indicate significant WF deviations.

As shown in Figure 1 and Figure S3, WF_air_ and WF_HV_ of most films deviate from WF_UHV_, i.e., the WF values of the “clean” films after outgassing in UHV conditions. Generally, WF_air_ is higher than WF_UHV_, with a WF difference of ΔWF_air-UHV_ in the range of 0.1–0.4 eV for donors and 0–0.26 eV for NFAs. Meanwhile, WF_HV_ is lower than WF_UHV_, with a WF difference of ΔWF_HV-UHV_ in the range −0.24–0 eV for donors and −0.27–0 eV for NFAs. The extent of WF deviation from UHV values varies across different materials, as summarized in Table S2. Note that most donors (except for PTO2) show a more prominent WF deviation in ambient air (|ΔWF_air-UHV_| ≥ |ΔWF_HV-UHV_|), while most acceptors (except for O-IDTBR) show a more prominent WF deviation in HV (|ΔWF_air-UHV_| ≤ |ΔWF_HV-UHV_|).

Generally, free-standing impurity-free or intrinsic OS films are thought to feature an *E*_F_ sitting midpoint of the gap separating the HOMO and LUMO levels. When brought into contact with a substrate, the *E*_F_ may either become pinned due to integer charge transfer between the OS film and substate at the interface or match the position of the substrate *E*_F_ in the absence of charge transfer (vacuum level alignment).^37^ This initial *E*_F_ position of the pristine OS film can be modified due to doping where p-doping will result in a deeper lying *E*_F_ (increased WF) regardless if the initial substrate energy level alignment was in a pinned mode or vacuum level alignment mode,^38^ and n-doping will yield a more shallow lying *E*_F_ (decreased WF). Furthermore, impurities can easily dope OS films during preparation or storage, accompanied by WF increasing (*E*_F_ moving down toward the HOMO level), showing a p-doping trend, or WF decreasing (*E*_F_ moving up toward the LUMO level), showing a n-doping trend. WF deviations observed in OS films under various atmospheric conditions, as measured by KP, suggests that the doping states of both donor and acceptor films are prevalently influenced by impurities (O_2_ or H_2_O) remaining in the films, resulting in shifts in the *E*_F_ or chemical potential. Namely, WF increases in air with coexistence of H_2_O and O_2_, indicating a p-doping trend, while WF decreases with H_2_O molecules left in films under the high vacuum of the KP chamber, suggesting a n-doping trend.

### 2.2 Reversible Doping Trend

**Figure 2:**
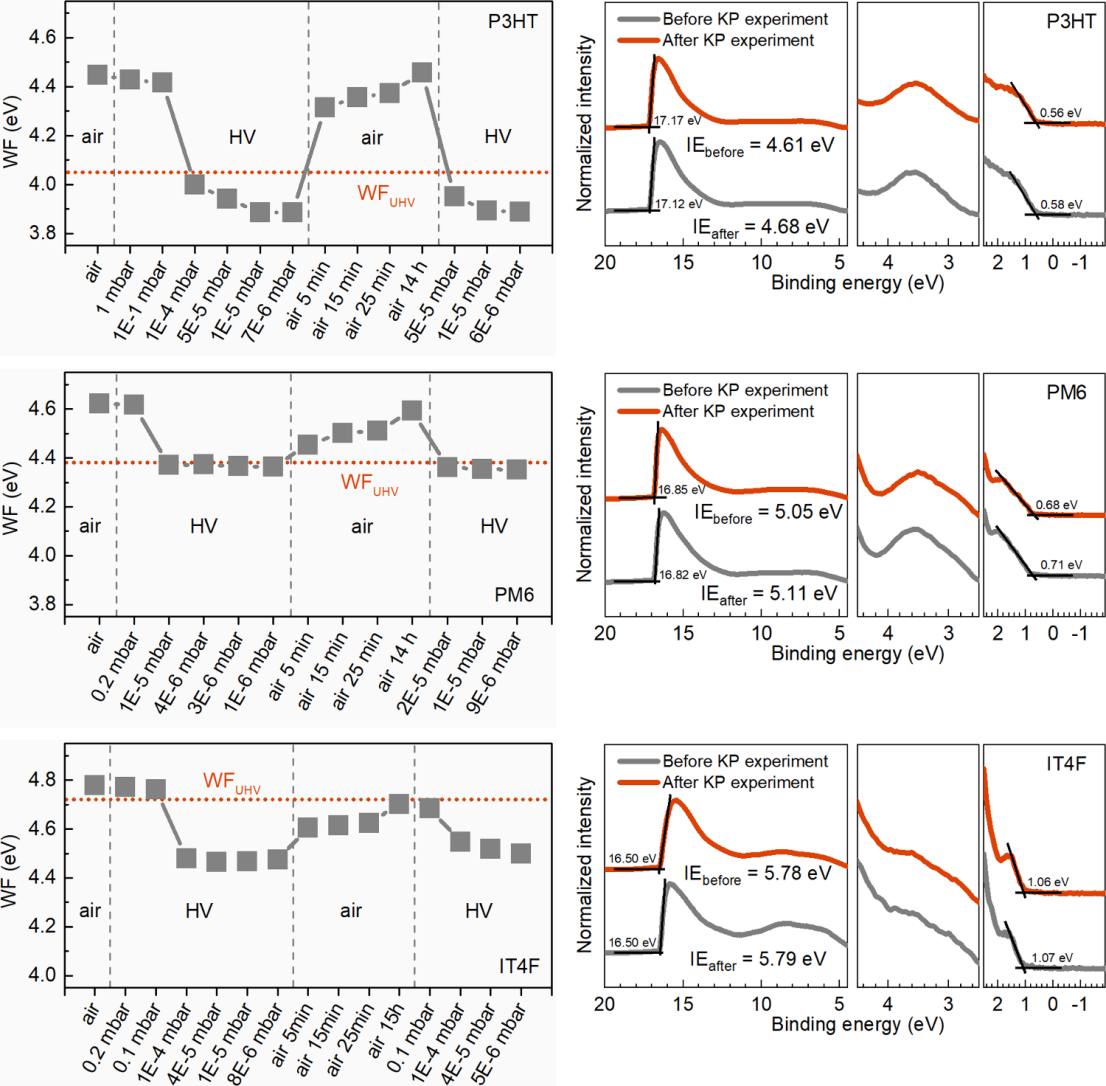
Left panel: Change in film work function during air/HV cycling in the dark. Right panel: UPS spectra of P3HT, PM6, and IT4F films before and after the KP measurement.

Aside from the doping effects, other factors like chemical degradation, surface dipoles, and thickness-dependent WF changes due to band bending at interfaces^39^ can affect WFs of films. To identify the main factor responsible for the observed WF shifts, the WF changes (CPD variations) of OS films are monitored in situ while cycling between air and HV (air/HV) in the KP chamber. Figure 2 shows the WF values of P3HT, PM6, and IT4F films under different atmospheric conditions. The valence electronic structures and ionization energies (IEs) of these films are also followed by UPS before and after KP experiments, respectively. All three materials show a similar trend of WF decrease from ambient air to a plateau under HV, but P3HT, PM6, and IT4F films exhibit distinct deviation patterns relative to WF_UHV_ measured by UPS. The equilibrated WF values of P3HT under both ambient air and HV conditions deviate from WF_UHV_, while PM6 shows a significant deviation from its WF_UHV_ only in ambient air. In contrast, IT4F exhibits a more pronounced WF deviation under HV conditions compared with ambient air. The deviation trends are consistent with the results in Figure 1. After the chamber is vented with air, the WFs of the films gradually increase and reach equilibrium after several hours, with the equilibrium values closely matching their initial values in ambient air. Upon remeasuring UPS after completing the full cycle of KP experiments, only a slight increase in IE, within the margin of error, is observed for P3HT and PM6, while no IE change is detected for the IT4F film. The valence electronic structures of the films remain unchanged compared to their state prior to the KP atmosphere-cycling experiments.

The reversible WF changes during air/HV cycling together with the unchanged valence electronic structures and stable IEs indicate that chemical degradation is not the main factor inducing the WF changes during this short-term air exposure under dark. It is also observed that the WF change from ambient air to the HV of an OS film is independent of relative humidity within the 20–40% range (Figure S5), suggesting that the OS film likely adsorbs a similar amount of H_2_O molecules regardless of humidity. This behavior may contribute to the relatively stable performance of OPVs processed or operated in ambient conditions with low humidity.^40−42^ By comparing the WFs of OS films with different thickness in different atmospheric conditions (Figure S6), no obvious WF dependence on film thickness is found. By monitoring the WF evolution of OS films with air exposure time starting from HV conditions, it is found that WFs of OS films change slowly with air exposure time. According to previous studies on the diffusion coefficients of O_2_ (∼10^–8^ cm^2^·s^–1^) and H_2_O (∼10^–10^ cm^2^·s^–1^) in OS films,^16,43^ these molecules can diffuse throughout the OS film of several tens of nm thick within a second.^17,21^ The slow WF change yet instantaneous diffusion of the O_2_/H_2_O into the bulk suggests that the WF shift of the OS film in air follows a thermal activated p-doping process, rather than a surface effect induced by surface physiosorbed O_2_ or H_2_O dipoles. Moreover, the WF change shows an exponential time dependence upon air exposure for these OS films (Figure S7), following the similar p-doping trend found by Maddalena et al.^20^ The reversible WF changes with minimum chemical damage during air/HV cycling are consistent with the reversible p-doping phenomenon induced by O_2_, as observed in many OS materials.^15,17,44,45^

### 2.3 *E*_F_ Shifts in Ambient Air (p-Doping Trend)

**Figure 3:**
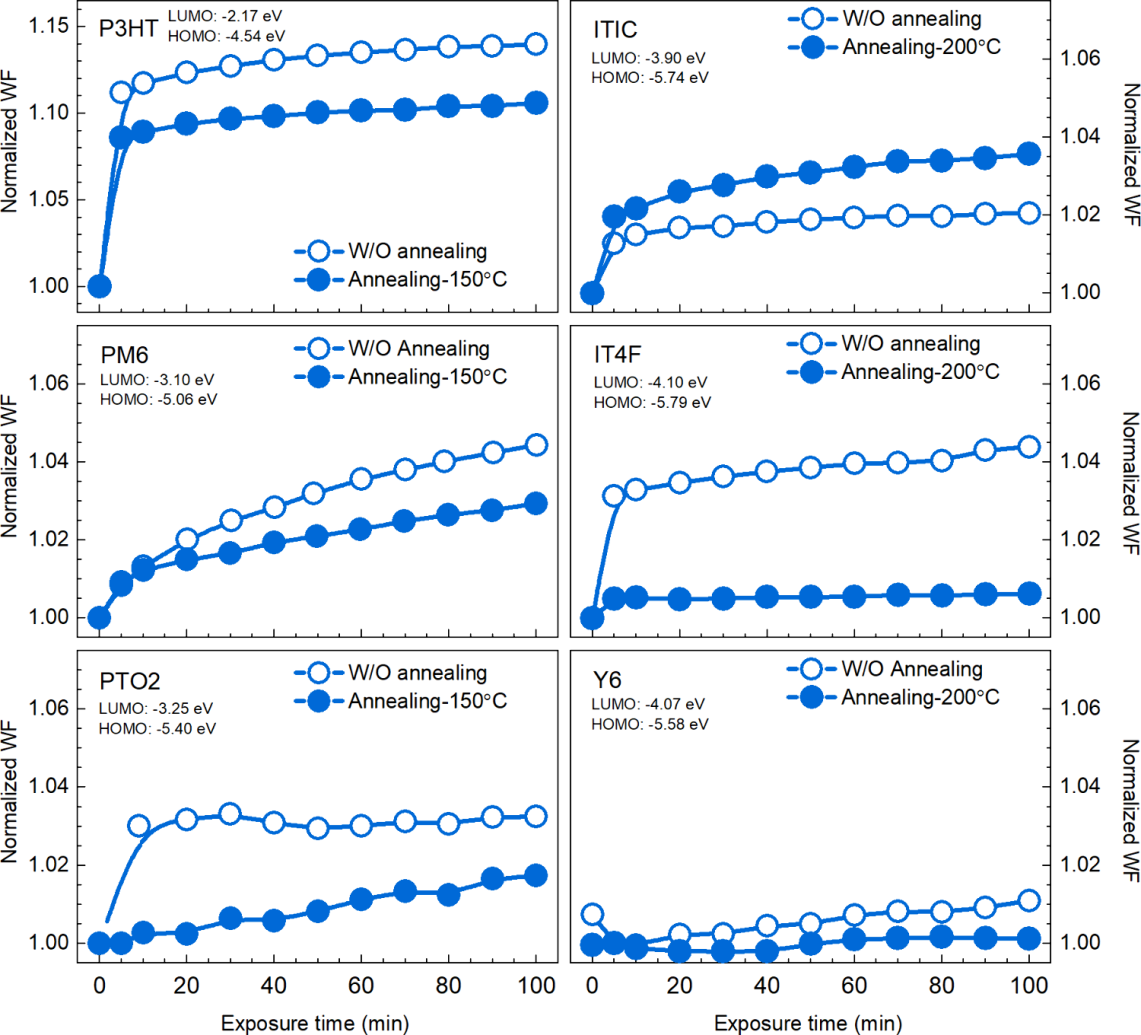
Normalized work function evolution with air exposure time in P3HT, PM6, PTO2, ITIC, IT4F, and Y6 films with or without (W/O) annealing. WFs at exposure time = 0 min are measured under HV conditions in the KP chamber of ∼1 × 10^–5^ mbar. All films are measured under dark. HOMO levels are measured by UPS, and LUMO values are abstracted from the literature, which are measured by inverse photoelectron spectroscopy (IPES).^50^

Since p-doping is a fundamental and crucial phenomenon that affects the properties of organic electronic devices, exploring the material-specific factors influencing the p-doping process (*E*_F_ downward shifting or WF increase) in ambient air would be beneficial for material design. Here, we choose several OSs with different energy levels (Figure S8) to follow their WF evolution with air exposure time. Figure 3 shows the WF evolution with air exposure time of three donors (P3HT, PM6, PTO2) and three NFAs (ITIC, IT4F, Y6) under dark. All films, nonannealed and annealed, feature a WF increase with air exposure time, displaying a typical p-doping trend as discussed before, but the size of the WF change differs significantly within the initial 100 min of air exposure. For both annealed and nonannealed donor films, P3HT, having the shallowest energy levels (LUMO = −2.17 eV, HOMO = −4.54 eV), shows the largest WF increase, while WF changes are smaller for the PM6 and PTO2 films that have deeper energy levels. For nonannealed NFAs, the sizes of WF change in the initial 100 min air exposure do not show obvious energy level dependence, but the annealed NFA films do. Annealed Y6 and IT4F films, with deeper LUMO levels, show smaller WF changes compared to the annealed ITIC film, whose LUMO level is approximately 0.2 eV shallower than those of Y6 and IT4F. Nonannealed IT4F films exhibit the largest WF change during the first 100 min of air exposure compared to the other two NFAs, despite having the deepest HOMO and LUMO levels (LUMO = −4.10 eV, HOMO = −5.79 eV). Notably, IT4F films also display more disordered morphology^46−48^ than ITIC and Y6 films, as shown in the near-edge X-ray absorption fine structure (NEXAFS) spectra (Figure S9, Table S3). This suggests that, in addition to energy level, film morphology plays a significant role in influencing the p-doping process in air. The effect of film morphology on the p-doping process can be observed by comparing the changes in WF from ambient air to HV conditions for annealed films versus nonannealed ones (Figure S10). All the annealed films show smaller WF changes than their nonannealed counterparts, which should be attributed to the more condensed and more ordered morphology obtained after thermal annealing,^49^ leaving less nanovoids for p-dopant adsorption in films.^24^

We note that p-doping in ambient air can occur under darkness, which means that photodoping through photoexcited LUMO electron transfer is not the only pathway for p-doping in ambient air, although light illumination is able to accelerate the p-doping process of OS films in ambient air. As shown in Figure S11, PM6 films show higher WF increasing rates under light illumination compared to those under dark, for both nonannealed and annealed films. IT4F and Y6 films, whether annealed or nonannealed, are less affected by light exposure. This can be attributed, on one hand, to their deep LUMO levels, which inhibit photodoping through LUMO electrons. On the other hand, their deep HOMO levels may facilitate the formation of n-doping sites, potentially competing with p-doping site formation and resulting in slower or even stable WF changes in the OS films. This will be discussed in greater detail in the following paragraphs.

**Figure 4:**
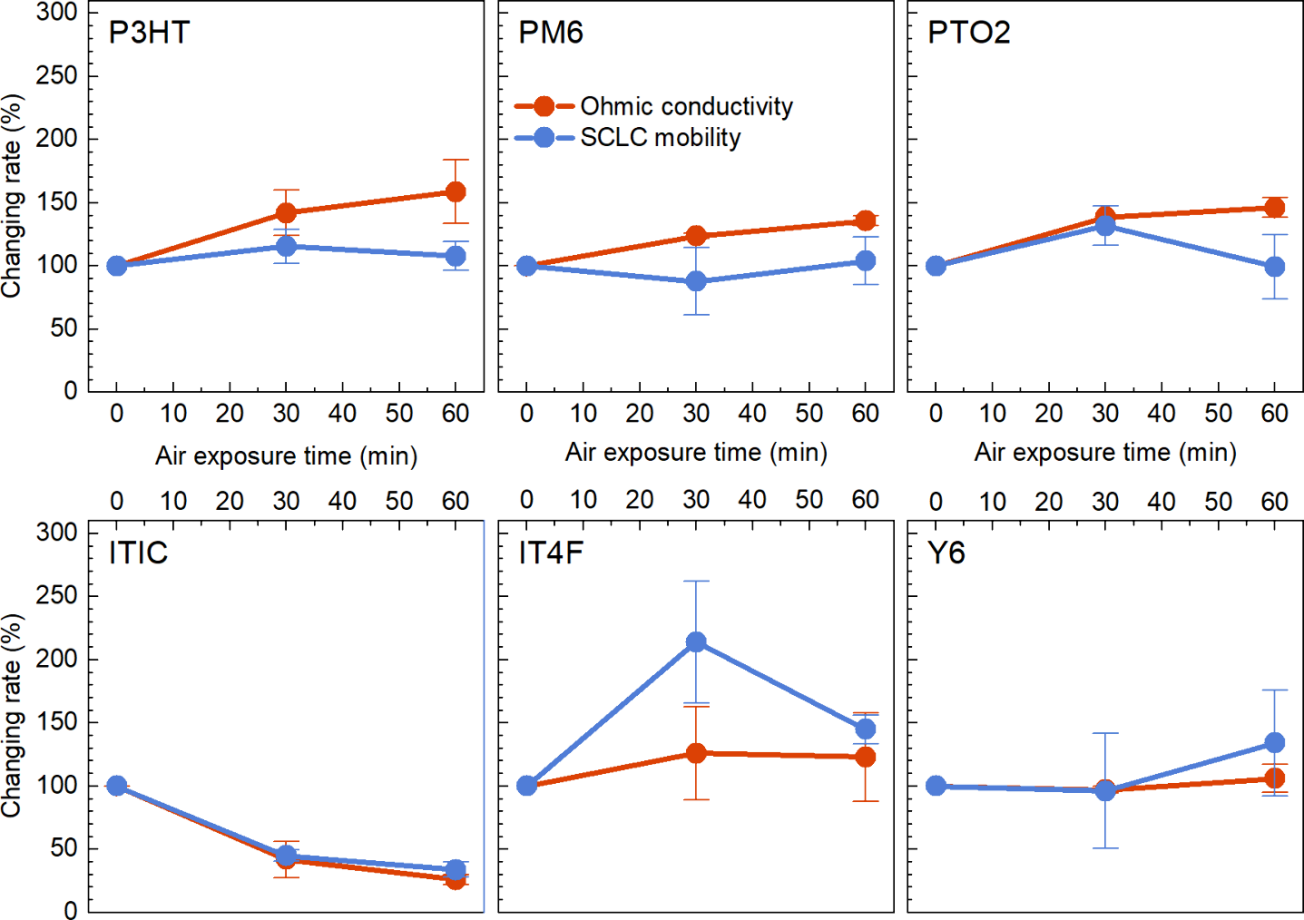
Changing rate of Ohmic conductivity and SCLC hole mobility in hole-only devices as a function of air exposure time for donors (P3HT, PM6, PTO2) and acceptors (ITIC, IT4F, Y6), with values referenced to glovebox conditions (0 min air exposure).

The p-doping trend can also be observed in hole-only devices where the conductivities and mobilities of materials under varying durations of air exposure are followed through the space-charge-limited-current (SCLC) method. Based on the measured current density–voltage (*J*–*V*) curves of hole-only devices (Figure S12), the Ohmic conductivities can be resolved from the slope = 1 region of the *J*–*V* curves on a double-log scale, and the SCLC mobilities are extracted from fits to the slope ≈2 region of the *J*–*V* curves using the Mott–Gurney law.^51,52^ For better comparison, Ohmic conductivity and SCLC mobility for each device are normalized at the values measured in a glovebox (0 min). As shown in Figure 4, donors (P3HT, PM6, PTO2) show an increasing trend in Ohmic conductivity, but the SCLC mobility does not change significantly during air exposure, suggesting that the increase in conductivity is more dependent on the increase of free charge carrier (the holes) concentration induced by O_2_ or O_2_/H_2_O complex in air, rather than an increase in mobility.

In contrast, there are no signs of the hole concentration increasing in acceptor films after air exposure since the Ohmic conductivity either shows the same decreasing trend as the mobility (ITIC) or remains constant (IT4F, Y6). This may indicate a different doping mechanism in ambient air for acceptors compared with those donors. The prominent mobility changes of acceptors after air exposure could be related to changes in film morphology or trap density after water or oxygen adsorption. Based on previous observations of the n-doping characteristics of acceptors under HV conditions—similar to glovebox conditions—and findings that water clusters act as hole traps in OS films with deep HOMOs,^23^ we suspect that the p-doping trend (observed as an increase in WF) in acceptors after air exposure may be due to two factors: the introduction of p-dopants (O_2_-related species) competing with pre-existing n-dopants (H_2_O) and changes in the dielectric environment following air exposure.

### 2.4 *E*_F_ Shifts in HV Conditions (n-Doping Trend)

To investigate a possible n-doping phenomenon in OS films, we focus on the deviation of WFs toward lower WF values in HV compared to those in UHV, especially for acceptors with deep HOMO levels. For convenience, we refer to the decrease in WF as an n-doping trend, although this phenomenon may also stem from destabilization of the frontier energy levels due to dielectric effects. First, we use the deviation of WF measured in HV (or air) from that measured in UHV, represented as |ΔWF_HV-UHV_| (or |ΔWF_air-UHV_|), as an indicator of the degree of n-doping (or p-doping) by residual n-dopants (or p-dopants) in films under various atmospheric conditions, and examine its relationship with the energy levels.

**Figure 5:**
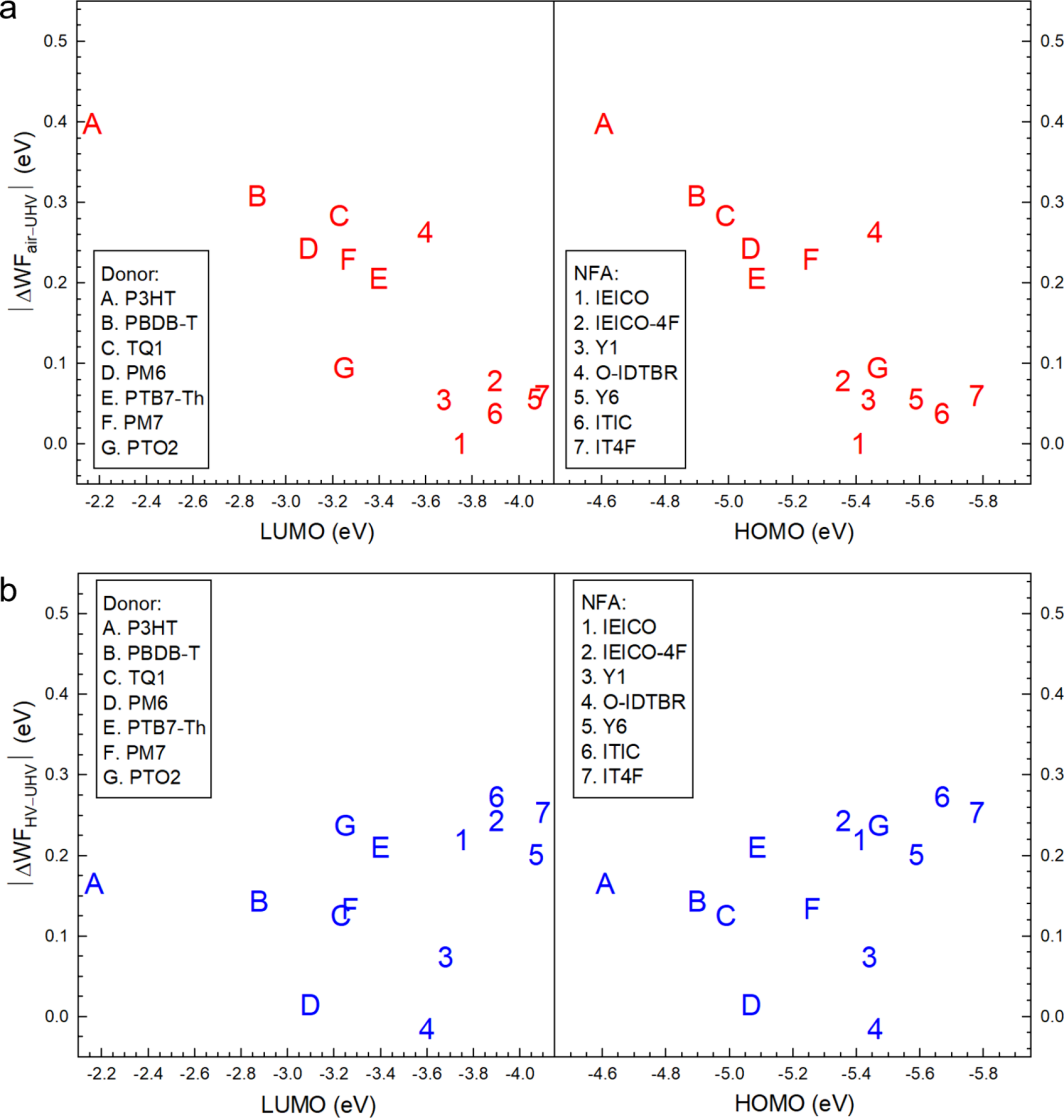
(a) The p-doping trend of OS films in ambient air (|ΔWF_air-UHV_|) and (b) the n-doping trend of OS films in HV (|ΔWF_HV-UHV_|), versus the LUMO or HOMO level of corresponding films. The error bar is ±0.07 eV for both |ΔWF_air-UHV_| and |ΔWF_HV-UHV_|. HOMO levels are measured by UPS, and LUMO values are abstracted from literature, which are measured by IPES.^50^

As presented in Figure 5a, |ΔWF_air-UHV_| (p-doping degree) decreases with increasing LUMO and HOMO levels. The lowest |ΔWF_air-UHV_| values are obtained for OSs with LUMO levels of around 3.8–4.0 eV and HOMO levels of around 5.4–5.8 eV. In contrast, the n-doping degrees (Figure 5b), inferred from |ΔWF_HV-UHV_| values, display a slight upward trend with increasing LUMO and HOMO levels, despite more variability across energy levels. Exceptions to this trend include PM6, Y1, and O-IDTBR, whose |ΔWF_HV-UHV_| values lie near the error margin. Data points deviating from the main doping trend may partly result from previously mentioned morphological variations or destabilization of molecular orbitals due to dielectric effects, as discussed later. Additionally, the relatively higher uncertainty in determining LUMO levels may also contribute to this scattering pattern.^50^

Our observations on WF shifts in ambient air are consistent with findings in the literature,^15,17,21,44,45^ where O_2_ or H_2_O/O_2_ complexes are regarded as the dominant p-dopants in films that increase the WF in ambient air. However, less attention has been paid to the n-doping trend in OS films, possibly due to the overwhelmingly dominant p-doping by the O_2_ or H_2_O/O_2_ complexes in ambient air, making the n-doping trend invisible. In our experiments, obvious n-doping trends are observed in most OS films kept under HV conditions, where H_2_O molecules are left as the most possible n-dopants.

**Figure 6:**
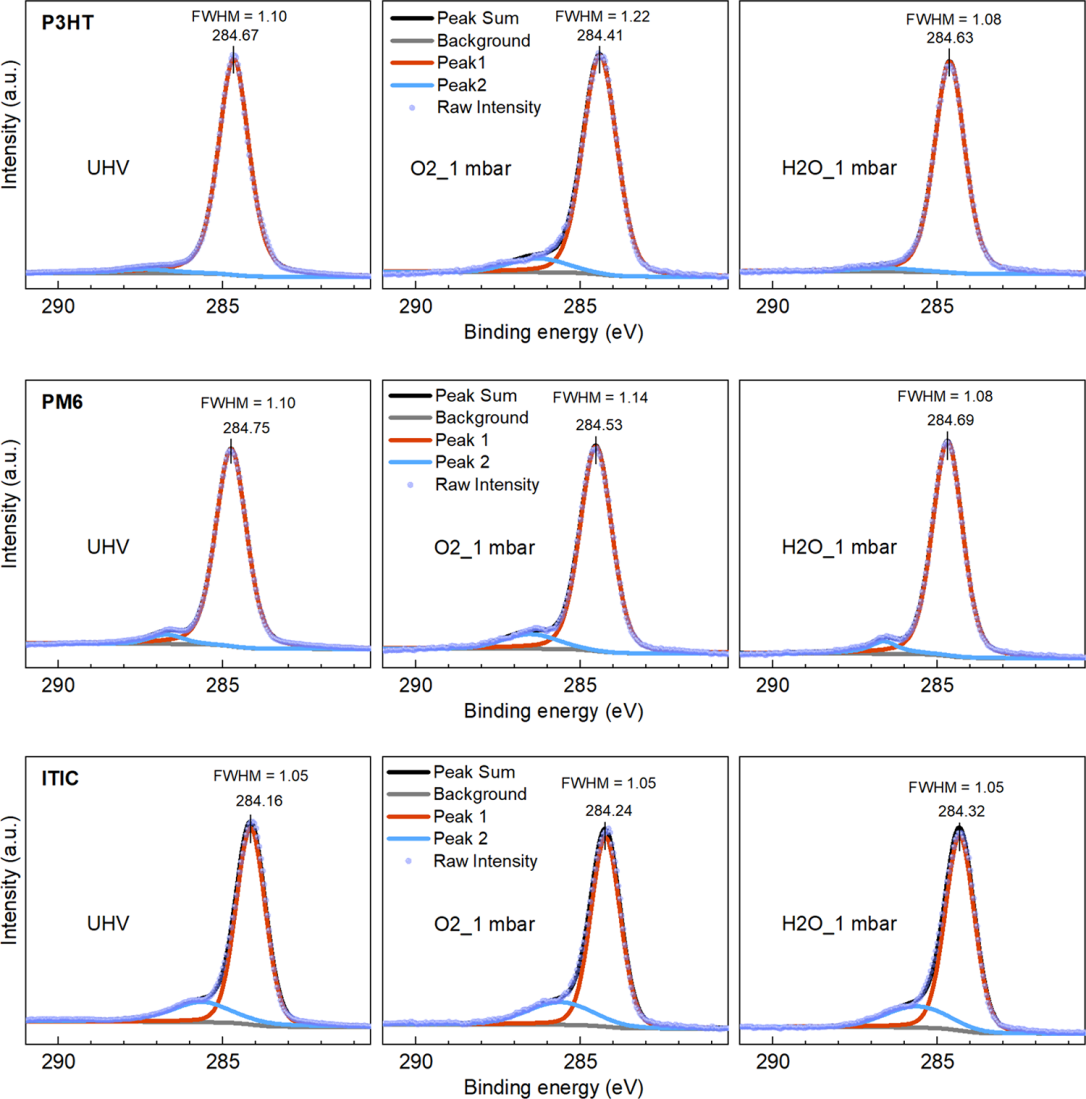
Impact of O_2_ and H_2_O vapor on the C 1s peaks of P3HT, PM6, and ITIC films under sequential atmospheric conditions: UHV → 1 mbar O_2_ → HV → 1 mbar H_2_O → HV. Only the conditions ‘UHV’, ‘O_2_ (1 mbar)’, and ‘H_2_O (1 mbar)’ are displayed.

To explore whether H_2_O molecules can induce an n-doping trend, we conducted NAP-XPS experiments to monitor in situ changes in the core-level structures of various OS films by sequentially changing the atmosphere. Initially, samples are measured under UHV followed by measurements in a near ambient pressure (NAP) cell^53^ with the atmospheric sequence ‘1 mbar O_2_→ HV → 1 mbar H_2_O → HV’, following the methodology of our previous work.^54^Figure 6 presents the C 1s peaks of P3HT, PM6, and ITIC films under UHV, O_2_, and H_2_O exposure. The complete C 1s spectra across all atmospheric conditions are provided in Figure S13, and the peak information has been summarized in Table S4.

In general, the C 1s spectra can be deconvoluted into two peaks. Peak 1 corresponds to carbon atoms in the environment of C=C–C and C=C–S. Peak 2 is for C–O, C=O (in PM6), and C≡N (in ITIC) components in OSs, in which the shakeup features are also included. In P3HT films exposed to 1 mbar of O_2_, the main peak (Peak 1) shifts to a binding energy 0.26 eV lower than in UHV, accompanied by broadening of the full width at half maximum (FWHM). Additionally, the area of Peak 2 (associated with oxygen-interacting carbons) increases in the O_2_ atmosphere, all indicating a p-doping trend. This effect persists even after evacuation to HV following O_2_ exposure (Figures S13 and S14), potentially due to oxidized states or residual, strongly adsorbed O_2_ on the films. These O_2_-associated structures are largely reduced upon introducing H_2_O vapor, demonstrating that H_2_O can counteract the p-doping effect induced by O_2_. PM6 shows a similar trend in C 1s peak shifting and broadening in an O_2_ atmosphere as P3HT. These changes are reversed upon returning to HV conditions, with the peak position shifting back to UHV values further after exposure to H_2_O. The C 1s peak shapes of PM6 under “HV after O_2_” and “H_2_O_1 mbar” conditions show minimal differences (Figure S14), which may suggest a weaker influence of water adsorption on carbon elements in PM6, consistent with the smaller water-related work function deviation observed in the KP measurements. Despite this, the mutual passivation effect of O_2_ and H_2_O in PM6 can also be observed from the evolution of O 1s peaks in Figure S15, where the shape changes of the O 1s peak induced by O_2_ exposure are reversed by H_2_O exposure.

In contrast to the donor materials P3HT and PM6, the acceptor ITIC remains relatively unaffected by O_2_ exposure but more sensitive to H_2_O exposure, which also aligns with findings from previous KP experiments. As shown in Figure 6 and Figures S13 and S14, the C 1s peak shape remains largely unchanged upon O_2_ exposure, exhibiting only a slight 0.08 eV shift to higher binding energy compared to that in UHV. Under H_2_O exposure, however, this peak shifts further to a higher binding energy of 284.32 eV. Additionally, the area of Peak 2 in ITIC remains mostly stable under O_2_ exposure but shows a slight decrease in an H_2_O atmosphere. A similar shift to higher binding energies is also observed in S 2p peaks (Figure S16), suggesting an n-doping trend. Moreover, significant peak broadening in the N 1s spectra occurs exclusively under water exposure (Figure S17), suggesting a likely interaction between water and the cyano (C≡N) group. This heightened water sensitivity is also observed in the Y6 films, as shown by the NAP-XPS spectra (Figure S18) for a Y6 film exposed sequentially to ‘UHV → 1 mbar H_2_O → HV’. Similar experiments on Y6 films have been reported previously.^54^

The increase in the oxygen-interacting carbon peak and the shift of the main peak to a lower binding energy in an O_2_ atmosphere indicate a typical p-doping effect. Interestingly, exposure to H_2_O reverses this p-doping, shifting the peak in the opposite direction, suggesting an n-doping role for H_2_O compared to the p-doping effect of O_2_. For ITIC, which has deeper energy levels, O_2_ exposure shows no clear p-doping trend, whereas H_2_O exposure induces a prominent n-doping effect, highlighting that a material’s tendency for p- or n-doping depends on its energy levels. This mutual passivation effect between H_2_O and O_2_ aligns with previous findings^22,25−27^ and suggests that reversible catalytic reactions, such as oxygen reduction (p-doping) and potential water oxidation (n-doping), may occur within these OSs. While oxygen reduction with O_2_ molecules in OSs has been widely documented,^11,55,56^ reports on water oxidation reactions in OSs are scarce. Given the n-doping potential of H_2_O molecules observed in our study, water oxidation reactions in OSs appear to be plausible.

### 2.5 Discussion

Based on our findings regarding the factors influencing the WF shift trends of OSs under different atmospheric conditions and the distinct roles of H_2_O and O_2_ molecules in interacting with OS films, we propose two scenarios to explain the WF shift phenomena in ambient air.

**Figure 7:**
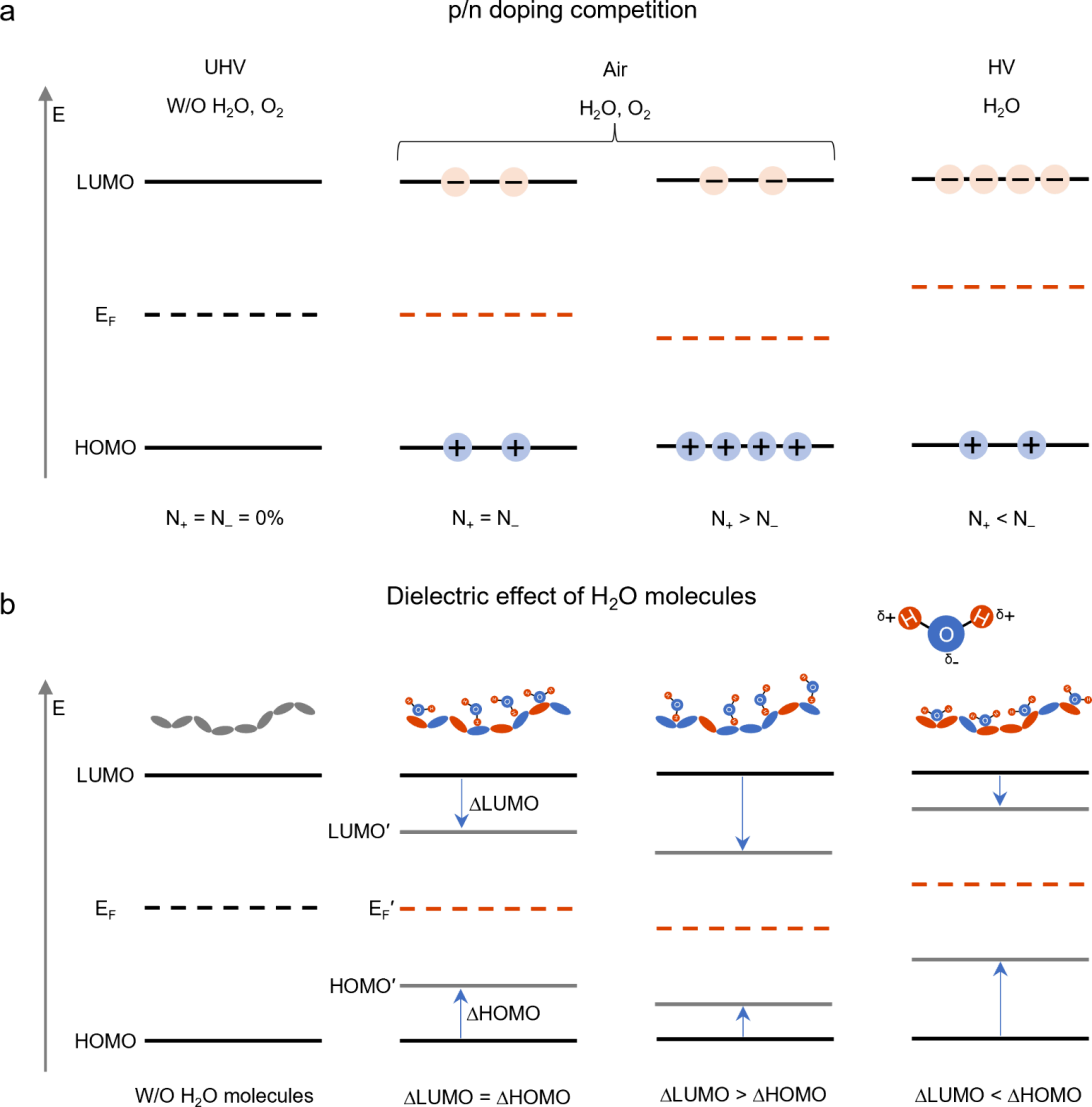
(a) Scenario 1: competition between p-doping and n-doping effect due to the changing of the relative concentration between the p-doped (N_+_) and n-doped (N_–_) sites in OSs; (b) Scenario 2: destabilization of energy levels due to dielectric effect of H_2_O molecules. LUMO′, HOMO′, and *E*_F_′ represent the energy levels after accounting for the dielectric effect of H_2_O molecules. ΔLUMO and ΔHOMO represent the shifts in the LUMO′ and HOMO′ levels relative to their original levels (LUMO, HOMO) in the absence (W/O) of dielectric effects from H_2_O molecules.

In the first scenario (Figure 7a), we observe a competition between p-doping and n-doping effects due to varying relative concentrations of p-doped sites (induced by O_2_ or H_2_O/O_2_ complexes) and n-doped sites (induced by H_2_O clusters). We hypothesize that the different WFs seen in OS films under various atmospheric conditions result from changes in the relative concentrations of O_2_ and H_2_O during the air/HV cycle. Moreover, given the same type and concentration of dopants, the energy required for an OS film to form p-doped or n-doped states varies, leading to an energy-level-dependent doping trend. Consequently, the competition between p-doping and n-doping reactions affects the relative concentration of p- and n-doped sites, causing the *E*_F_ to move up (the WF decreasing) or down (the WF increasing) relative to the vacuum level. This scenario is supported by evidence that the doping trends in OS films by H_2_O and O_2_ molecules depend on film morphology and energy levels, which determine the available adsorption sites and potential differences for charge transfer, respectively.

The second scenario (Figure 7b) involves the destabilization of energy levels due to the dielectric effect of H_2_O molecules. The HOMO and LUMO energy levels of a molecule are influenced by the dielectric environment surrounding it as variations in permittivity can stabilize or destabilize these electronic states. H_2_O molecules, being polar, can screen both positive and negative charges on OS molecules. This polarity stabilizes OS polarons, resulting in a larger electron affinity and smaller ionization energy, i.e., moving the LUMO and HOMO levels into the gap.^27,57^ Different OSs have varying affinities for H_2_O molecules, especially NFA-based systems with alternating push–pull structures. The *E*_F_ can deviate from its pristine state if H_2_O molecules affect the HOMO and LUMO levels unequally. For instance, an “n-doping” trend occurs when the HOMO level moves up more than the LUMO level moves down, raising the *E*_F_ relative to the pristine OS. This may help explain the “n-doping trend” observed in certain OSs, where the negative pole of the H_2_O molecules is thought to preferentially interact with electron-deficient segments that dominate the HOMO level. Conversely, the “p-doping trend” in OSs could result from H_2_O molecules orienting their positive pole toward electron-rich segments that dominate the LUMO level.

Although both scenarios can explain the *E*_F_ shift rules induced by O_2_ and H_2_O molecules, the actual mechanism is likely more complex. The final *E*_F_ in the OS film is probably influenced by both mechanisms acting simultaneously. Changes in energy levels due to H_2_O’s dielectric effect can affect the chemical potential difference between OSs and dopants, altering doping reactions, which in turn modify the electrostatic potentials on OS molecules, affecting H_2_O’s dielectric effects. Therefore, to achieve stable WF in OS films in ambient air, one should select materials with appropriate energy levels within a certain safe range, as suggested by Blom et al.,^22,23^ and aim to remove nanovoids and condense films to reduce dopant concentration and minimize H_2_O’s dielectric effects, aligning with trap suppression methods proposed by Kemerink et al.^24^

## 3 Conclusions

In summary, a general trend of *E*_F_ shift of OS materials in ambient air is found and the factors to affect the *E*_F_ shift are discussed. By comparing the WF values in UHV, most OSs show a p-doping trend of higher WFs in ambient air with coexistence of O_2_ or H_2_O molecules but show a n-doping trend of lower WFs in HV conditions with more H_2_O molecules remaining in the films. This *E*_F_ shift phenomenon is reversible without obvious changes in chemical structures during the experiments. Further KP experiments of WF dependence on air exposure time show that the p-doping trend is dependent on two main factors: energy levels and film morphology. Deeper energy levels as well as a more condensed film morphology will help to decrease the p-doping rates of OS materials in ambient air. However, deeper energy levels also enhance the n-doping trend in OSs, as indicated by the NAP-XPS results. Two scenarios are proposed to explain the mechanism of “p-doping” and “n-doping” phenomena with the *E*_F_ shift direction as an indicator: (1) the competition between p-doping and n-doping effect results in the changing of the relative concentration between the p-doped sites (by O_2_ or H_2_O/O_2_ complexes) and n-doped sites (by H_2_O clusters); (2) the destabilization of energy levels due to the dielectric effect of H_2_O molecules. Based on these mechanisms, a safe energy window for trap-free OSs still exists in ambient air. However, the threshold energy levels that define this window become more diffuse and narrower compared to cases with trace amounts of O_2_ or H_2_O, due to the intricate interactions between OSs, O_2_, H_2_O, and their resulting complexes. Consequently, caution should be exerted on the atmosphere during the WF determination of OS materials, and *E*_F_ shifts of OSs should be considered during the ambient-processing and ambient-operation of devices. In addition, the possible catalytic activities of these OSs in oxygen reduction and water oxidation reactions inferred from this work suggest their promising application in catalysis.

## 4 Experimental Section

### Materials and Film Fabrication

All of the organic semiconductor (OS) materials were obtained from 1-Material Inc. The OS materials were dissolved into a solution of 3–10 mg/mL in chloroform (CF), except for Y1, which was dissolved in chlorobenzene (CB), then the solution was spin-coated on the ITO substrates (WF: 4.6–4.7 eV) with a spin speed of 3000 rpm. Film thickness is measured through a depth profiler (Dektak XT Bruker). Polymer donor films have thicknesses of approximately 20, 40, and 100 nm for 3, 6, and 10 mg/mL solutions, respectively. Small molecule acceptor films reach about 20 and 40 nm for 6 and 10 mg/mL solutions, respectively. All the film preparation procedures including the thermal annealing were carried out in a N_2_-filled glovebox with O_2_ < 1 ppm and H_2_O < 1 ppm.

### Kelvin Probe (KP)

KP measurements were carried out with a KP6500 digital Kelvin probe provided by McAllister Technical Services. The Kelvin probe provided the contact potential difference (CPD) between the sample and the reference probe, with a resolution of around 10 meV. The reference probe was made of standard stainless steel with a diameter of 3 mm, and its work function was calibrated by previously UPS characterized substrates with stable work functions in both ambient air and high vacuum, such as Al/AlO_*x*_, HOPG, and ITO. The calibration routine was performed before and after the measurement series to avoid the experimental errors brought by probe contamination during the experiments. During the measurements, the vibration frequency and amplitude were set to 100 Hz and 20 au, respectively, and the gradient of the peak-to-peak versus backing potential was set to 0.1. The final CPD value for each sample was obtained from the averaged values of 20 independent measurements. KP measurements were carried out under both ambient air and vacuum with a pressure of ≥10^–6^ mbar. Samples were transferred and measured under dark. For the WF evolution with time experiment under light, a small LED light panel was placed under the sample to provide light illuminance.

### Ultraviolet Photoelectron Spectroscopy (UPS)

UPS measurements were performed under a base pressure lower than 1 × 10^–9^ mbar in a dedicated home-designed and built spectrometer. Low-intensity monochromatized He I radiation (*hv* = 21.22 eV) with an energy resolution of 50 meV and a large spot-size was used in the experiments. Work functions and ionization energies (IEs) of samples are derived from the same way as we reported before.^48^ All the measurements were calibrated by referencing to the Fermi level of the Ar^+^ ion sputter-cleaned gold foil. Radiation damage was carefully examined, and no damage was detected. All measurements were carried out under dark.

### Near-Edge X-ray Absorption Fine Structure (NEXAFS) Spectroscopy

NEXAFS measurements were carried out at the FlexPES (Flexible Photoelectron Spectroscopy) beamline at the MAX IV synchrotron radiation facility in Lund, Sweden. A defocused beam with horizontally linear polarization within a energy range of 40–1500 eV was utilized, and the energy resolution is about 20 meV at photon energies close to the C K-edge. Angle-dependent NEXAFS spectra were collected in total electron yield (TEY) detection mode with sample drain current and in partial electron yield (PEY) mode with a multichannel plate simultaneously.

### Near-Ambient Pressure X-ray Photoelectron Spectroscopy (NAP-XPS)

NAP-XPS measurements were performed at the SPECIES beamline at MAX IV. The beamline is equipped with an APPES end station, which allows surface-sensitive characterization of solid-vapour/gas-liquid interfaces at a pressure up to 10–20 mbar in the energy range of 30–1500 eV. XPS measurements were conducted on each sample following these atmospheric sequences: UHV → 1 mbar O_2_ → HV → 1 mbar H_2_O → HV. Measurements under UHV conditions were recorded at pressures below 10^–10^ mbar in the analysis chamber. High-vacuum (HV) measurements, performed at approximately 1 × 10^–5^ mbar, were carried out in a near-ambient pressure (NAP) cell. Both oxygen and water exposure experiments were conducted in the NAP cell, maintained at a base pressure of 1 mbar. During the XPS measurements, the photon energy 545 eV for N 1s, 435 eV for C 1s, and 310 eV for S 2p were used, respectively, with a pass energy of 50 eV and slit width of 50 um. In all cases, a series of spectra were recorded at different time intervals, different exposure times, and different spots of the film under synchrotron radiation to avoid beam damage. For clarity of presentation, only one representative data set per sample environment is shown.

### Device Fabrication and Measurements

Hole-only devices with a structure of an ITO/PEDOT:PSS/OS film (70–120 nm)/MoO_3_ (5 nm)/Ag (100 nm) were fabricated. The OS film was spin-coated on top of the PEDOT:PSS film from chloroform without the annealing process. After that, MoO_3_ and Ag contacts were evaporated in sequence under a pressure of 1 × 10^–6^ mbar. The current density versus voltage (*J*–*V*) curves of devices were measured by a Keithley 2400 in a glovebox first and then in ambient air with different exposure times of 30 and 60 min.

## Supporting Information Available

Figure S1: Chemical structures of donor and acceptor materials used in this work; Figure S2: Calibration of probe work function (WF) by substrates; Figure S3: e·CPD value of a sample measured in air or high vacuum versus its WF value measured under UHV; Figure S4: Stability assessment of the ITO substrate during UPS measurements; Table S1: Assessment of WF stability in ITO substrates under UV exposure during UPS measurement; Table S2: Summary of contact potential difference values and the corresponding WF values measured in air or high vacuum; Figure S5: Work function changes from ambient air to high vacuum conditions under different relative humidity; Figure S6: WF evolution with air exposure time for PM6 and Y6 films and WFs of PM6 and Y6 films with different thicknesses measured in air, high vacuum, and ultrahigh vacuum; Figure S7: WF evolution versus air exposure time for P3HT, PM6, and IT4F films; Figure S8: Energy levels of P3HT, PM6, PTO2, ITIC, IT4F, and Y6 films; Figure S9: Angular dependence of C K-edge NEXAFS spectra in total electron yield detection mode for PM6, ITIC, IT4F, and Y6 films; Table S3: Orientation determination of films from NEXAFS; Figure S10: Work function changes from ambient air to high vacuum conditions for films with or without annealing; Figure S11: The effect of annealing and light illumination (white LED) on the work function evolution with air exposure time in PM6, IT4F, and Y6 films; Figure S12: Experimental (dots) and fitted (lines) current density and slope versus voltage curves of hole-only devices; Figure S13: Evolution of the C1s peaks for P3HT, PM6, and ITIC films under sequential atmospheric conditions; Figure S14: Normalized C1s peaks of P3HT, PM6, and ITIC films under sequential atmospheric conditions; Figure S15: Normalized O1s peaks for PM6 under sequential atmospheric conditions; Figure S16: The influence of O_2_ and H_2_O vapor on the S2p peak of P3HT, PM6, and ITIC film; Figure S17. Normalized N1s peaks for ITIC under sequential atmospheric conditions; Figure S18: Normalized C1s, S2p, O1s, and N1s peaks for the Y6 film under UHV conditions and after exposure to 1 mbar H_2_O; Table S4: Summary of peak information of P3HT, PM6, and ITIC films under different atmospheric conditions from NAP-XPS spectra (PDF)
