# Supplementary material for: Fermi Level Shifts of Organic Semiconductor Films in Ambient Air
Source: ACS Appl Mater Interfaces. 2025 Jan 8;17(3):5153–64. doi: 10.1021/acsami.4c13674 (PMC11759101; doi:10.1021/acsami.4c13674)
Supplement: Supplementary file 1 — am4c13674_si_001.pdf [file am4c13674_si_001.pdf]

Supporting Information

Fermi level shifts of organic semiconductor films in ambient air

[1]*Xian'e Li**, [1,2]*Qilun Zhang*, [1]*Yongzhen Chen*, [1]*Xianjie Liu*, [1]*Slawomir Braun, and* [1,2]*Mats Fahlman**

[1]Laboratory of Organic Electronics, Department of Science and Technology (ITN), Linköping University, Norrköping SE-60174, Sweden.
[2]Wallenberg Wood Science Center, Department of Science and Technology (ITN), Linköping University, SE-60174, Norrköping, Sweden

* Correspondence to: xiane.li@liu.se (X.L.); mats.fahlman@liu.se (M.F.)

1. Supplementary Notes

Supplementary Note 1. Precautions when comparing WFs by UPS and KP

The differences in work function measured by KP and UPS are not solely attributed to the differences between ambient and vacuum conditions. This caution arises because UPS and KP rely on fundamentally different principles. UPS measures ionization energy by ejecting electrons using ultraviolet light in vacuum conditions, therefore, photon-irradiation-induced surface photovoltage, charging, or beam damage during measurements can lead to unreliable values. In our UPS measurements, monochromatized and defocused UV beam with a very low photon flux is utilized, thus these artifacts are largely avoided. In addition, calibration is a crucial issue for both UPS and KP. Normally, UPS spectra are calibrated by referencing the Fermi level of Ar^{+} ion sputter-cleaned gold foil. However, for KP measurement, calibration becomes trickier, since KP measures the contact potential difference between a reference electrode and the sample surface, often influenced by factors such as surface cleanliness, adsorbates, or environmental conditions, especially in ambient settings. Therefore, both the reference probe and the calibration substrates should have stable WFs in both air and vacuum. Additionally, the substrates used for calibration should also show stable WFs under UPS measurement. In this work, a stainless-steel reference probe with minimum contaminant

adsorption and stable WF in both air and vacuum is applied, and the probe WF is calibrated against several substrates with highly stable WFs, showing only minor variations (< 0.1 eV) from ambient air to high vacuum. This approach averages individual reference deviations, enhancing calibration reliability and accuracy. The substrates used for calibration in this work are the freshly cleaved surface of highly ordered pyrolytic graphite (HOPG), aluminum with a native oxide layer (Al/AlO_x) and indium tin oxide (ITO). However, it is found in some literature that ITO substrates show instable WFs under UV light during UPS measurements. To evaluate this, we measured the CPD of the ITO substrate before and after exposure to UHV in the UPS chamber, both with and without UV illumination. Results are summarized in **Table S1**. The WF varied by less than 0.1 eV (within the UPS measurement error margin) in both cases. We also assessed the stability of the ITO substrate during UPS measurements by recording UPS spectra of the ITO substrate at 5-minute intervals. No spectral shifts were observed throughout the measurement period, as shown in **Figure S4**. Our results differ from previous studies probably due to the low-dose, defocused UV source in our custom UPS setup, affirming ITO's stability as a reliable reference material in our calibration process. This method enables us to compare the WF differences under various ambient conditions based solely on the samples themselves, minimizing any influence from the reference probe. Other systematic errors from KP measurements, such as different probe-sample distance, also have been avoided by keeping the same measurement settings for each sample (see in the Experimental Methods).

Supplementary Note 2. The calibration of Kelvin Probe

Compared to photoelectron emission spectroscopy (UPS, XPS), Kelvin probe is more sensitive to the minor surface potential or WF changes of various organic or inorganic semiconductor films under both ambient atmosphere and low-to-high vacuum pressure ($\geq 10^{-6}$ mbar),[1–4] which enables us to monitor the WF changes of donors and NFAs during air/vacuum/air circulation in a more convenient way. However, KP measurements only provide the contact potential

difference (CPD) between the measured sample and the reference probe, thus a reference probe with minimum contaminant adsorption and stable WF in both air and vacuum should be applied, such as the stainless steel probe used in this work.[5] Besides, the probe WF is calibrated against a freshly cleaved surface of highly ordered pyrolytic graphite (HOPG), indium tin oxide (ITO) and aluminum with a native oxide layer (Al/AlO_x), whose CPD values are quite stable with only minor changes (0.008 V for Al/AlO_x, 0.02 ~ 0.03 V for ITO, 0.07 V for HOPG) from ambient air to high vacuum (**Figure S2**). The measurement error for e·CPD value from the KP experiment is ±0.01 eV (Y-error in **Figure S2**), while the experimental error for the UPS measurement is ±0.05 eV (X-error in **Figure S2**). A calibration line with a slope of -1 is fitted with the data points from those stable WF substrates when plotting the e·CPD values measured by KP in vacuum against their corresponding WF values measured in UHV (WF_{UHV}) by UPS (**Figure S2**). The WF of the stainless-steel reference probe (WF_{Ref}) is determined to be 4.5 eV from the intercept of the fitted line (where CPD = 0 V), with a standard error of ±0.05 eV. Then the translation between WF values and CPD values can be achieved by this simple formula WF = 4.5 eV - e·CPD. Similarly, the WF deviation of a sample measured in air (WF_{Air}) or high vacuum (WF_{HV}) compared to its WF measured in UHV, namely $\Delta WF_{Air\text{-}UHV} = WF_{Air} - WF_{UHV} = WF_{Ref} - e\cdot CPD_{Air} - WF_{UHV}$, and $\Delta WF_{HV\text{-}UHV} = WF_{HV} - WF_{UHV} = WF_{Ref} - e\cdot CPD_{HV} - WF_{UHV}$ should have an overall standard error of $\sqrt{0.05^2 + 0.01^2 + 0.05^2} \approx 0.07$ eV. The 95% confidence band of the calibration line establishes the confidence interval for the WF deviations of each material measured in air or high vacuum compared to their values in UHV. Data points outside this region indicate significant WF deviations.

Supplementary Note 3. The translation between CPD and WF values

For better comparison, CPD values of films in air (CPD_{Air}) or in high vacuum (CPD_{HV}) are plotted against their WF values measured in UHV (WF_{UHV}) in **Figure S3**, which enables the

direct comparison among CPD_{Air}, CPD_{HV}, and CPD_{UHV} (translated from WF_{UHV} values through the dashed calibration line in **Figure S2**, **Supplementary Note 2**) along the y-axis. For example, the WF of P3HT film measured by UPS is 4.05 eV corresponding to CPD_{UHV} = 0.45 V, while the CPD value drops to 0.05 V (WF increases to 4.45 eV) when P3HT is exposed in ambient air, or ascends to 0.62 V (WF decreases to 3.88 eV) when P3HT is kept in the KP chamber with a vacuum pressure around 4 magnitude order higher than in the UPS analysis chamber. The CPD values and corresponding WF values of these films in different atmospheres (air, HV, UHV) are summarized in **Table S2**.

2. Supporting Figures and Tables

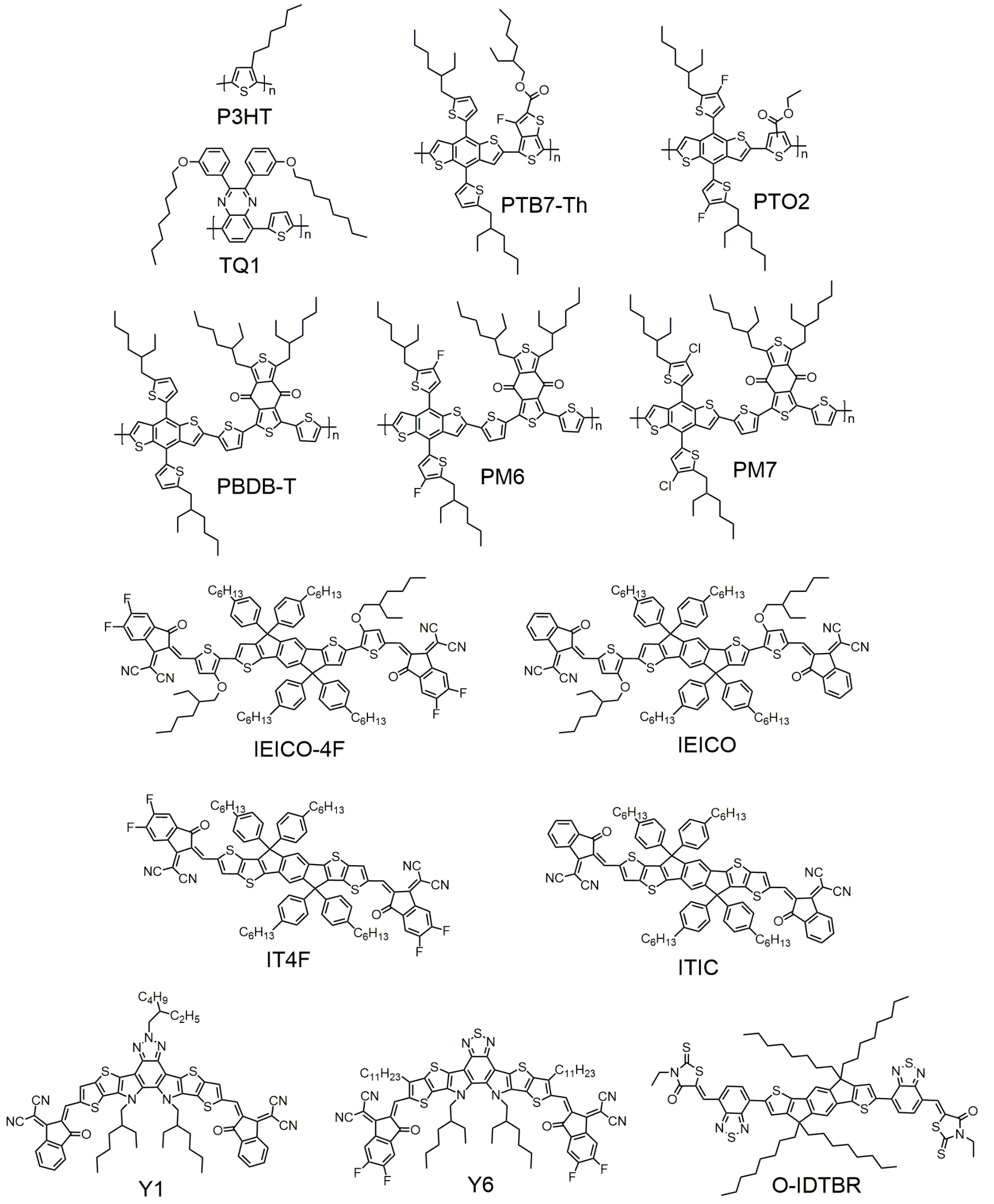

Figure S1. Chemical structures of donor and acceptor materials used in this work.

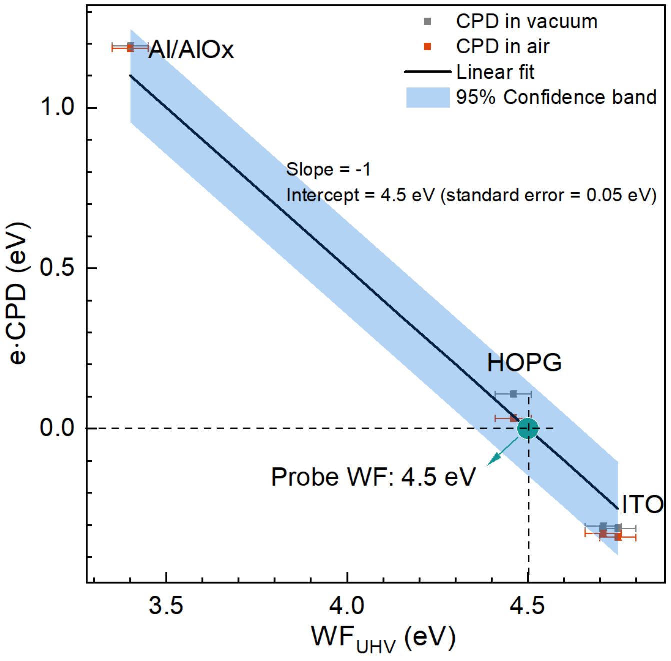

Figure S2. Calibration of probe work function (WF) by substrates (Al/AlO_x, ITO, HOPG) with stable WFs in air and vacuum. A fitting line with a slope of -1 (the black solid calibration line) is drawn among those data points of samples whose contact potential difference (e·CPD) values measured by Kelvin probe in vacuum are plotted against their WF values measured by ultraviolet spectroscopy (WF_{UHV}) in ultra-high vacuum. The WF of the reference probe is determined to be 4.5 eV from the intercept of the fitted line (where e·CPD = 0 eV), with a standard error of 0.05 eV. The 95% confidence band of this calibration line is also shown.

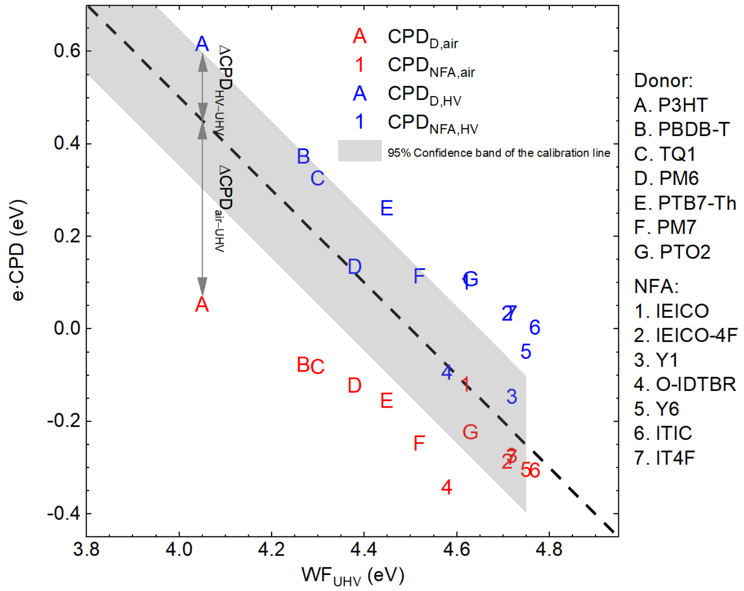

Figure S3. e·CPD value of a sample measured in air (red dots) or high vacuum (blue dots) by Kelvin probe (KP) versus its work function value measured in ultra-high vacuum by ultraviolet photoelectron spectroscopy (WF_{UHV}). The calibration line (dashed line) determined from Figure S2 serves as a guide for translation from e·CPD values (y-axis) to absolute WF values (x-axis), e.g., e·CPD = 0 eV corresponding to WF = 4.5 eV. The 95% confidence band of the calibration line establishes the confidence interval for the WF deviations of each material measured in air or high vacuum compared to their values in UHV. Data points outside this region indicate significant WF deviations.

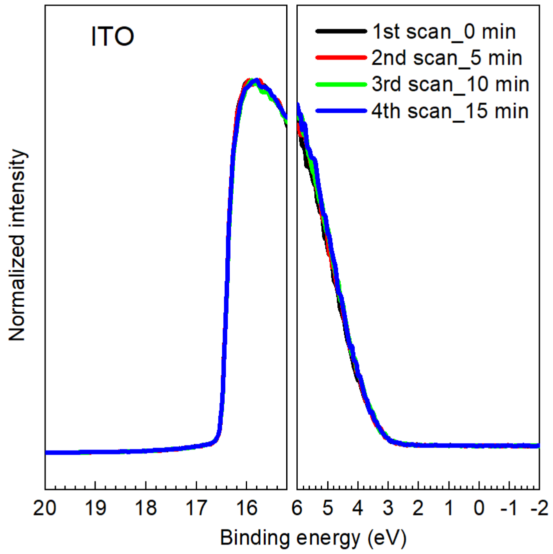

Figure S4. Stability assessment of the ITO substrate during UPS measurements, with UPS spectra recorded at 5-minute intervals. No spectral shifts were observed over the measurement period.

Table S1. Assessment of WF stability in ITO substrates under UV exposure during UPS measurement.

Measurement Step	Description	CPD (V)	WF (eV)
KP in air (As Received)	Initial measurement as received	-0.1895	4.69
No Measurement in UPS Chamber	Held in UPS analysis chamber (UHV) for 15 min without UV exposure (UV shutter off)	--	--
KP in air (After UHV Exposure)	After 15 min in UPS analysis chamber (UHV) without UV exposure	-0.1161	4.62
UPS Measurement	UPS measurement under UV light exposure for ~15 min	--	4.69
KP in air (Post-UPS Measurement)	After UPS measurement under UV exposure	-0.0900	4.59

Table S2. Summary of contact potential difference (CPD) values measured in air or high vacuum (HV) by Kelvin probe, and their corresponding work function (WF) values translated from the calibration line in Figure S2 (or by the formula WF = 4.5 eV – e·CPD), which are denoted as CPD_{air} (WF_{air}) and CPD_{HV} (WF_{HV}), respectively. WF_{UHV} values are obtained by ultraviolet spectroscopy (UPS) in ultrahigh vacuum (UHV), and the corresponding CPD values (CPD_{UHV}) are also translated from the calibration line with the same formula. The CPD (WF) difference between films in UHV and air is denoted as $\Delta CPD_{air\text{-}UHV}$ ($\Delta WF_{air\text{-}UHV}$), and the CPD (WF) difference between films in HV and UHV is denoted as $\Delta CPD_{HV\text{-}UHV}$ ($\Delta WF_{HV\text{-}UHV}$).

OS films	CPD_{air} (V)	CPD_{HV} (V)	CPD_{UHV} (V)	WF_{air} (eV)	WF_{HV} (eV)	WF_{UHV} (eV)	$\Delta CPD_{air\text{-}UHV}$ (V)	$\Delta CPD_{HV\text{-}UHV}$ (V)	$\Delta WF_{air\text{-}UHV}$ (eV)	$\Delta WF_{HV\text{-}UHV}$ (eV)
Donor										
P3HT	0.05	0.62	0.45	4.45	3.88	4.05	−0.40	0.17	0.40	−0.17
PBDB-T	−0.08	0.37	0.23	4.58	4.13	4.27	−0.31	0.14	0.31	−0.14
TQ1	−0.08	0.32	0.20	4.58	4.18	4.30	−0.28	0.12	0.28	−0.12
PM6	−0.12	0.13	0.12	4.62	4.37	4.38	−0.24	0.01	0.24	−0.01
PTB7-Th	−0.16	0.26	0.05	4.66	4.24	4.45	−0.21	0.21	0.21	−0.21
PM7	−0.25	0.11	−0.02	4.75	4.39	4.52	−0.23	0.13	0.23	−0.13
PTO2	−0.22	0.11	−0.13	4.72	4.39	4.63	−0.09	0.24	0.09	−0.24
Acceptor										
IEICO	−0.12	0.10	−0.12	4.62	4.40	4.62	0.00	0.22	0.00	−0.22
IEICO-4F	−0.29	0.03	−0.21	4.79	4.47	4.71	−0.08	0.24	0.08	−0.24
Y1	−0.27	−0.15	−0.22	4.77	4.65	4.72	−0.05	0.07	0.05	−0.07
O-IDTBR	−0.34	−0.10	−0.08	4.84	4.60	4.58	−0.26	−0.02	0.26	0.02
Y6	−0.31	−0.05	−0.25	4.81	4.55	4.75	−0.06	0.20	0.06	−0.20
ITIC	−0.31	0.00	−0.27	4.81	4.50	4.77	−0.04	0.27	0.04	−0.27
IT4F	-0.28	0.03	-0.22	4.78	4.47	4.72	-0.06	0.25	0.06	-0.25

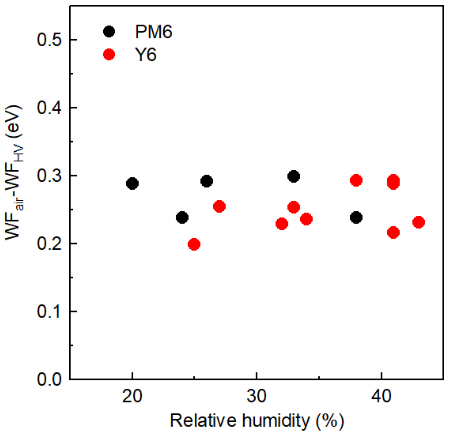

Figure S5. Work function changes from ambient air to high vacuum (HV) conditions for films under different relative humidity.

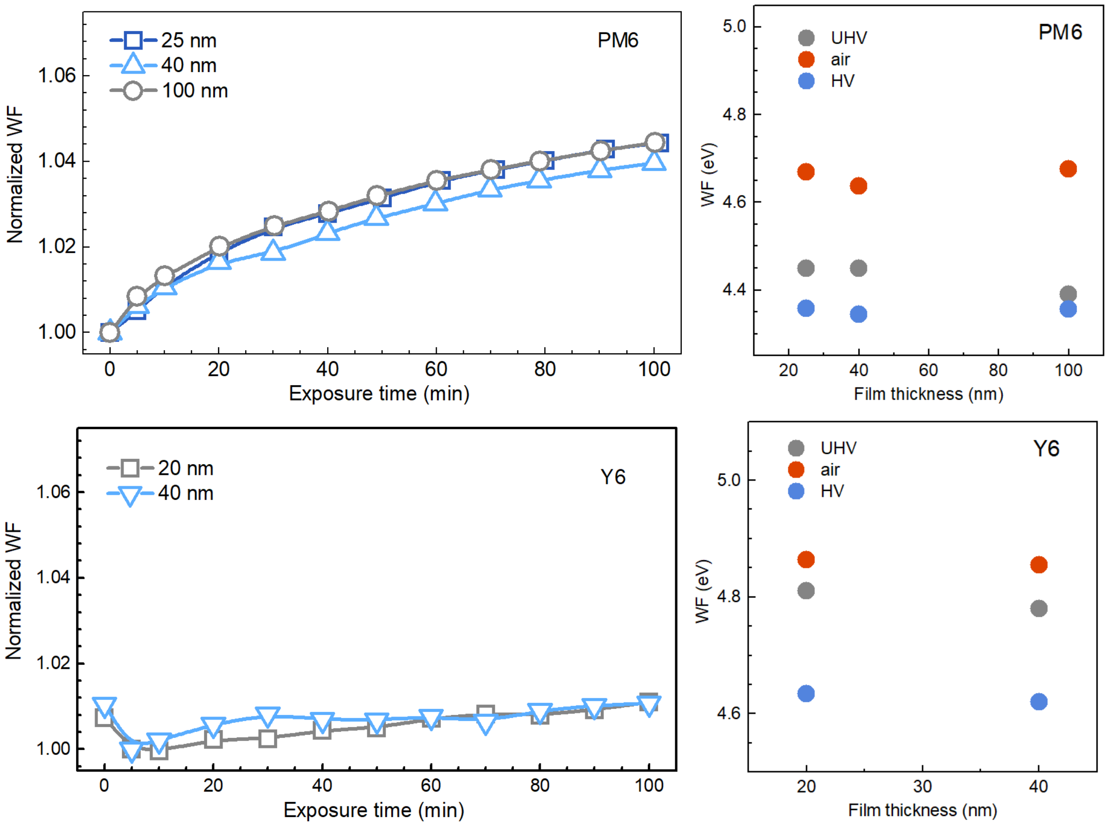

Figure S6. (Left) WF evolution with air exposure time for PM6, Y6 films of different thickness after venting the Kelvin probe chamber with air. (Right) WFs of PM6 and Y6 films with different thickness measured in air, high vacuum (HV), and ultrahigh vacuum (UHV), respectively.

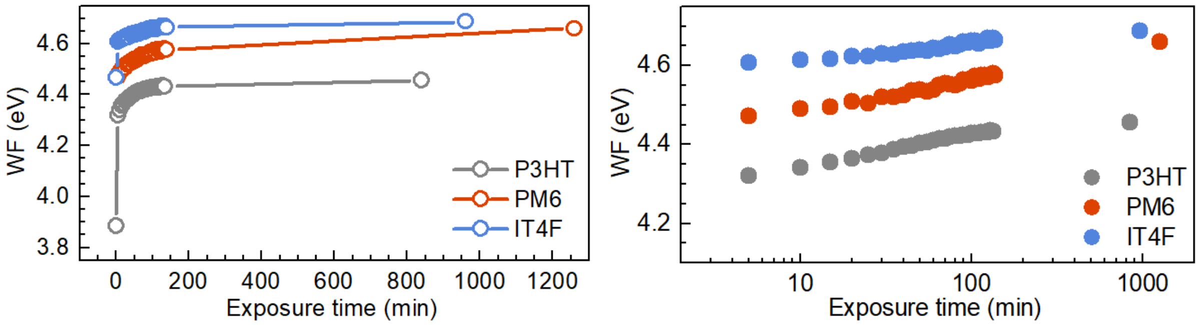

Figure S7. WF evolution versus air exposure time for P3HT, PM6 and IT4F films.

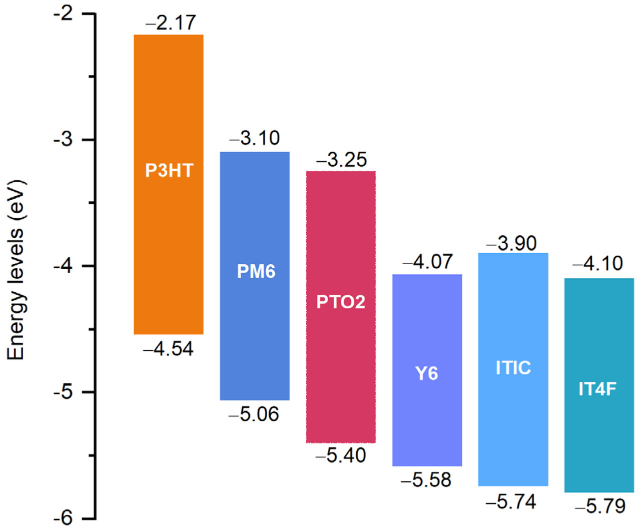

Figure S8. Energy levels of P3HT, PM6, PTO2, ITIC, IT4F and Y6 films. HOMO levels are measured by UPS, and LUMO values are abstracted from literature, which are measured by inverse photoelectron spectroscopy (IPES).[6]

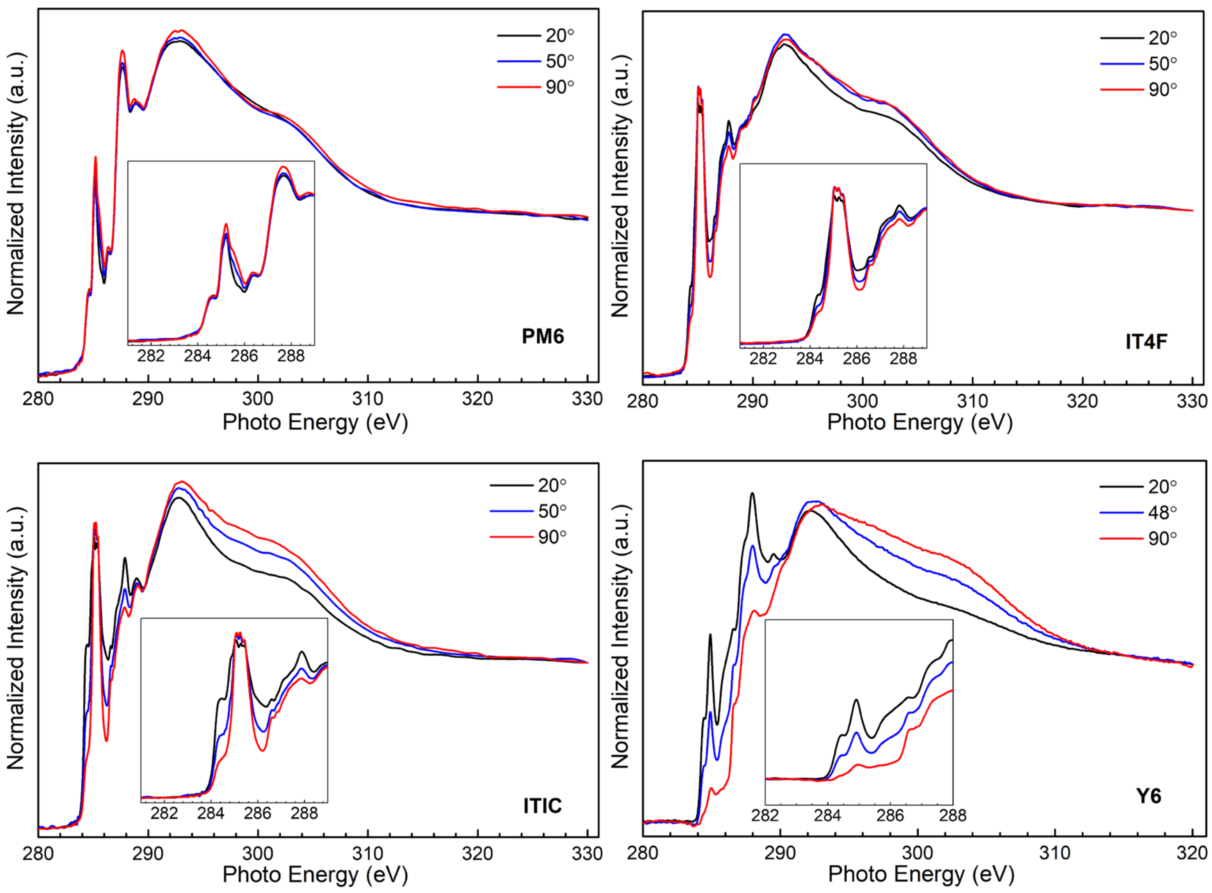

Figure S9. Angular dependence of C K-edge NEXAFS spectra in total electron yield (TEY) detection mode for PM6, ITIC, IT4F, Y6 films, with X-ray beam at 90° (normal incident), 20° (grazing incident) related to the sample surface. Portions of the spectra in this figure are reproduced from ref 7. Available under a CC-BY 4.0 license. Copyright [2022] Xian'e Li et al.[7]

Table S3 Orientation determination of films from NEXAFS.

Films	Angular dependence of C 1s → C=C π* transition peak intensity		Orientation suggested from NEXAFS
	Shoulder peak (284.4 eV)	Main peak (285 eV)	
PM6	No angular dependence	No angular dependence	No preferential orientation or tilted roughly at magic angle (≈ 54.7°)
ITIC	Angular dependence<br>Intensity (20° > 50° > 90°)	No angular dependence	Preferential face-on orientation
IT4F	Weak angular dependence<br>Intensity (20° > 50° > 90°)	No angular dependence	Preferential face-on orientation but more disordered
Y6	Angular dependence<br>Intensity (20° > 50° > 90°)	Angular dependence<br>Intensity (20° > 50° > 90°)	Preferential face-on orientation

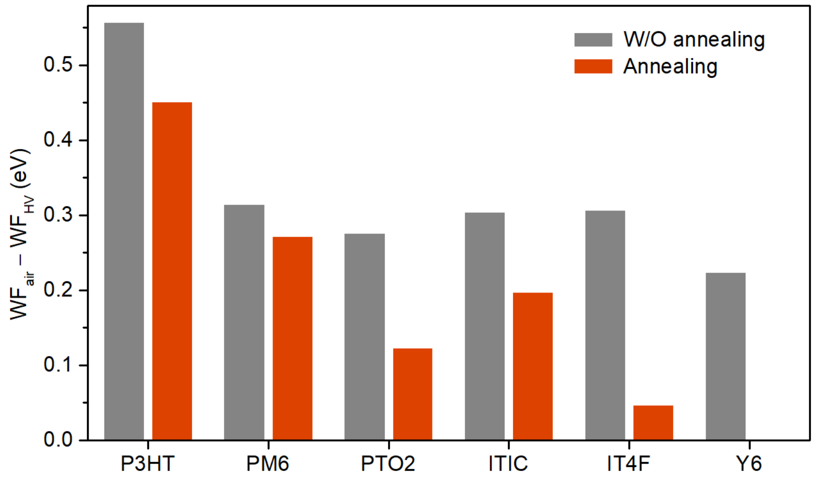

Figure S10. Work function changes from ambient air to high vacuum (HV) conditions for films with or without (W/O) annealing.

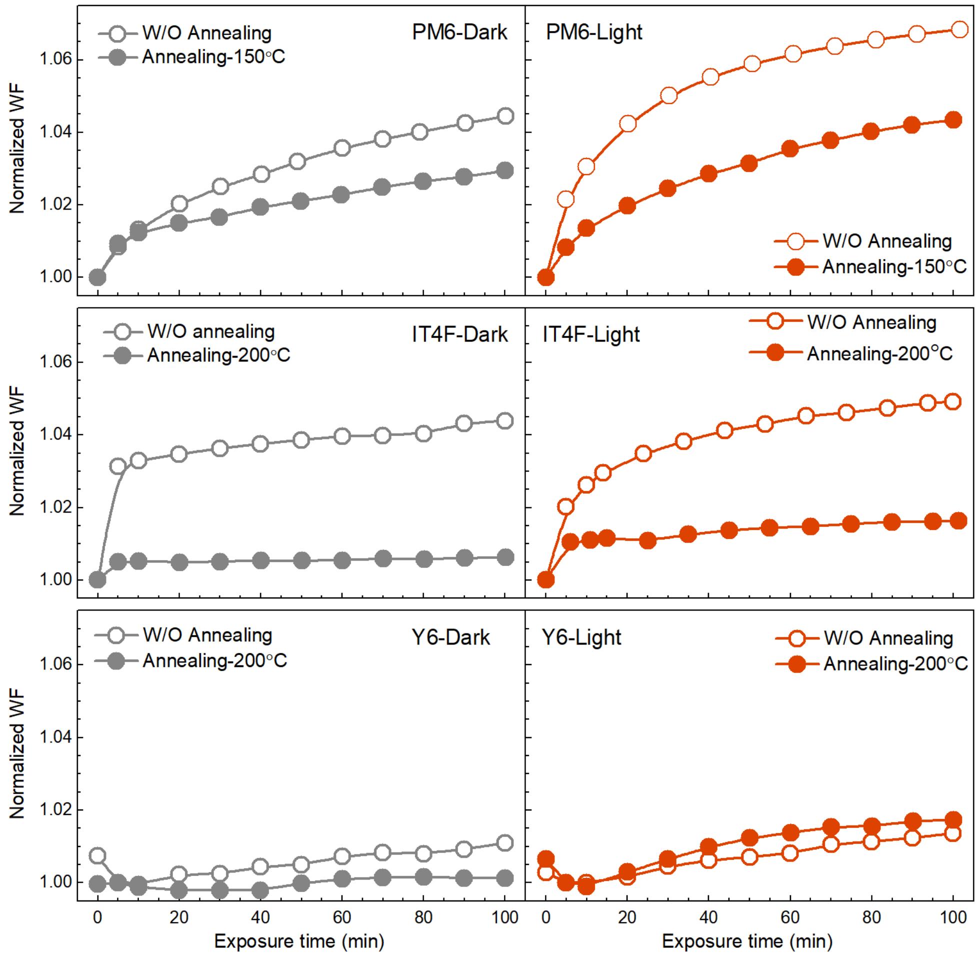

Figure S11. The effect of annealing and light illumination (white LED) on the work function evolution with air exposure time in PM6, IT4F, and Y6 films. WFs at exposure time = 0 min are measured at HV conditions in KP chamber of ~1×10^{-5} mbar.

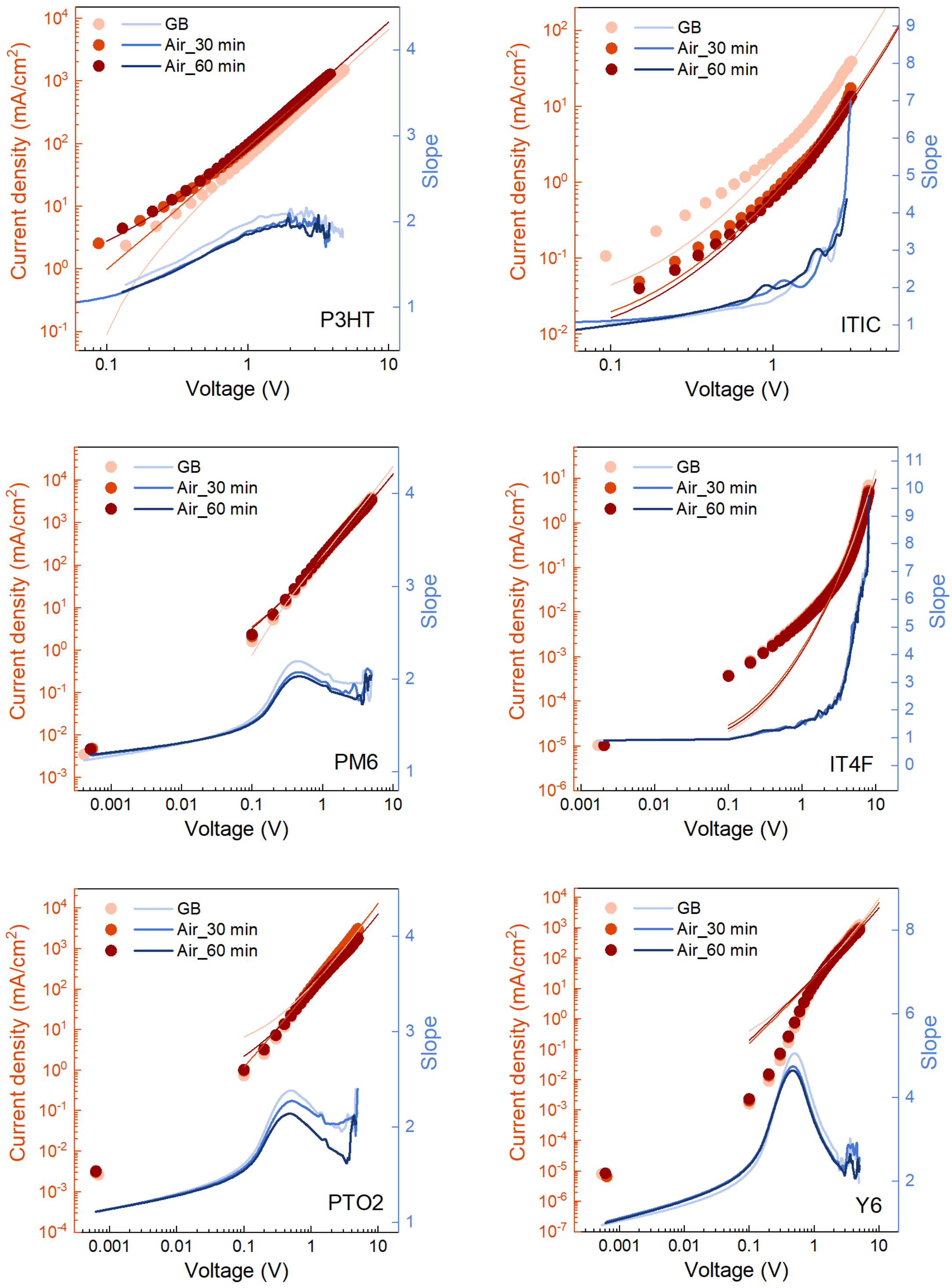

Figure S12. Experimental (dots) and fitted (lines) current density (red) and slope (blue) versus voltage curves of hole-only devices in an N_2-filled glovebox (GB) or in ambient air with different exposure time.

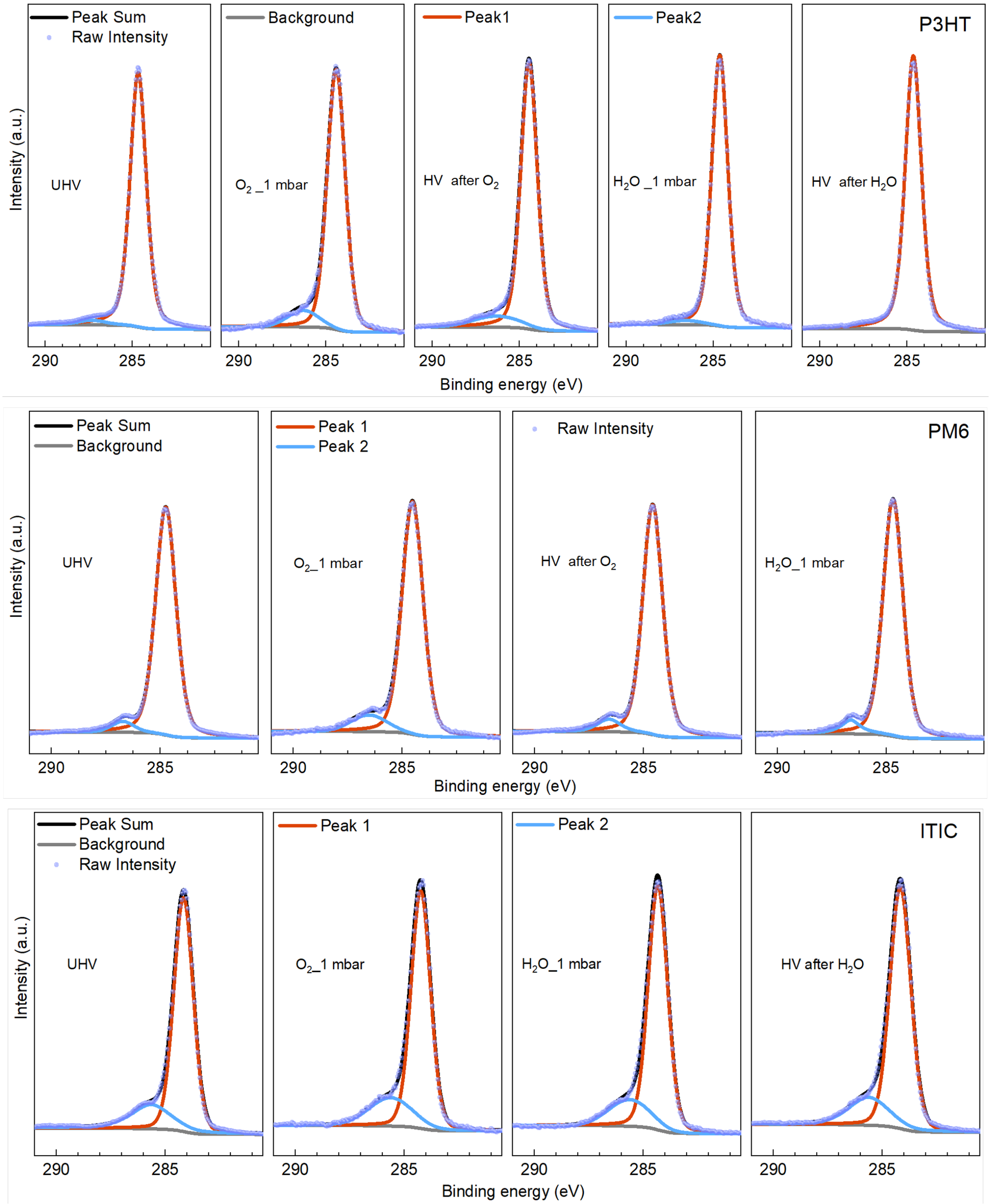

Figure S13. Evolution of the C1s peaks for P3HT, PM6, and ITIC films under sequential atmospheric conditions: UHV → 1 mbar O_2 → HV → 1 mbar H_2O → HV. The spectra for HV following H_2O exposure (HV after H_2O) in PM6 and HV following O_2 exposure (HV after O_2) in ITIC were not recorded due to limited beamline time.

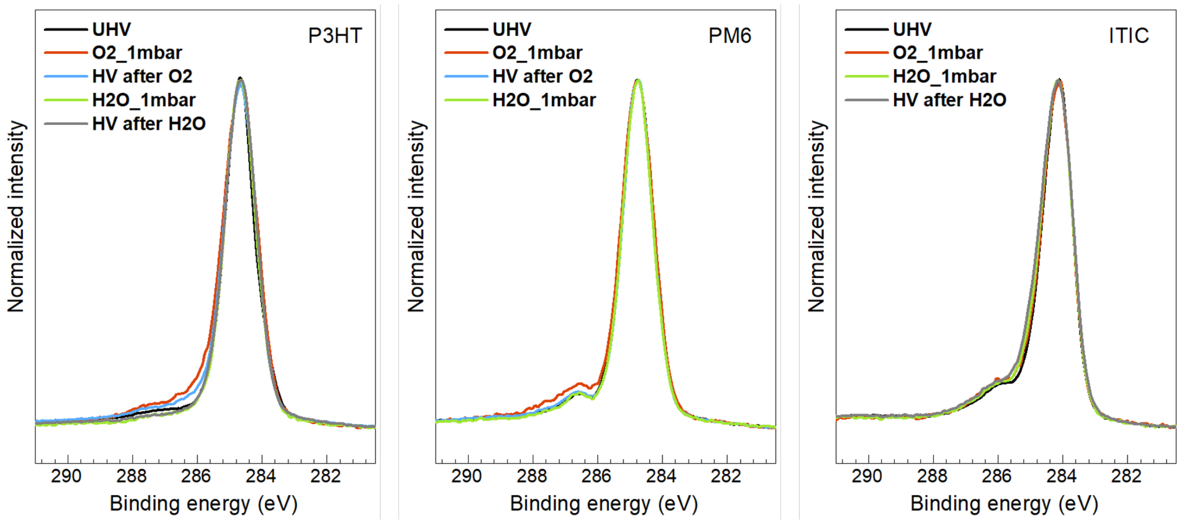

Figure S14. Normalized C1s peaks of P3HT, PM6, and ITIC films under sequential atmospheric conditions: UHV → 1 mbar O_2 → HV → 1 mbar H_2O → HV. Peaks are aligned to the same binding energy for improved comparison. The spectra for HV following H_2O exposure (HV after H_2O) in PM6 and HV following O_2 exposure (HV after O_2) in ITIC were not recorded due to limited beamline time.

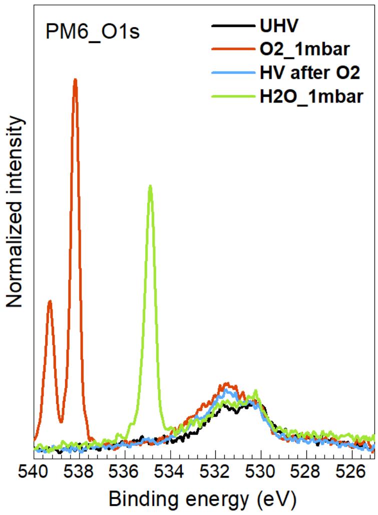

Figure S15. Normalized O1s peaks for PM6 under sequential atmospheric conditions: UHV → 1 mbar O_2 → HV → 1 mbar H_2O → HV. Peaks are aligned to the same binding energy for improved comparison. The spectra for HV following H_2O exposure (HV after H_2O) in PM6 were not recorded due to limited beamline time.

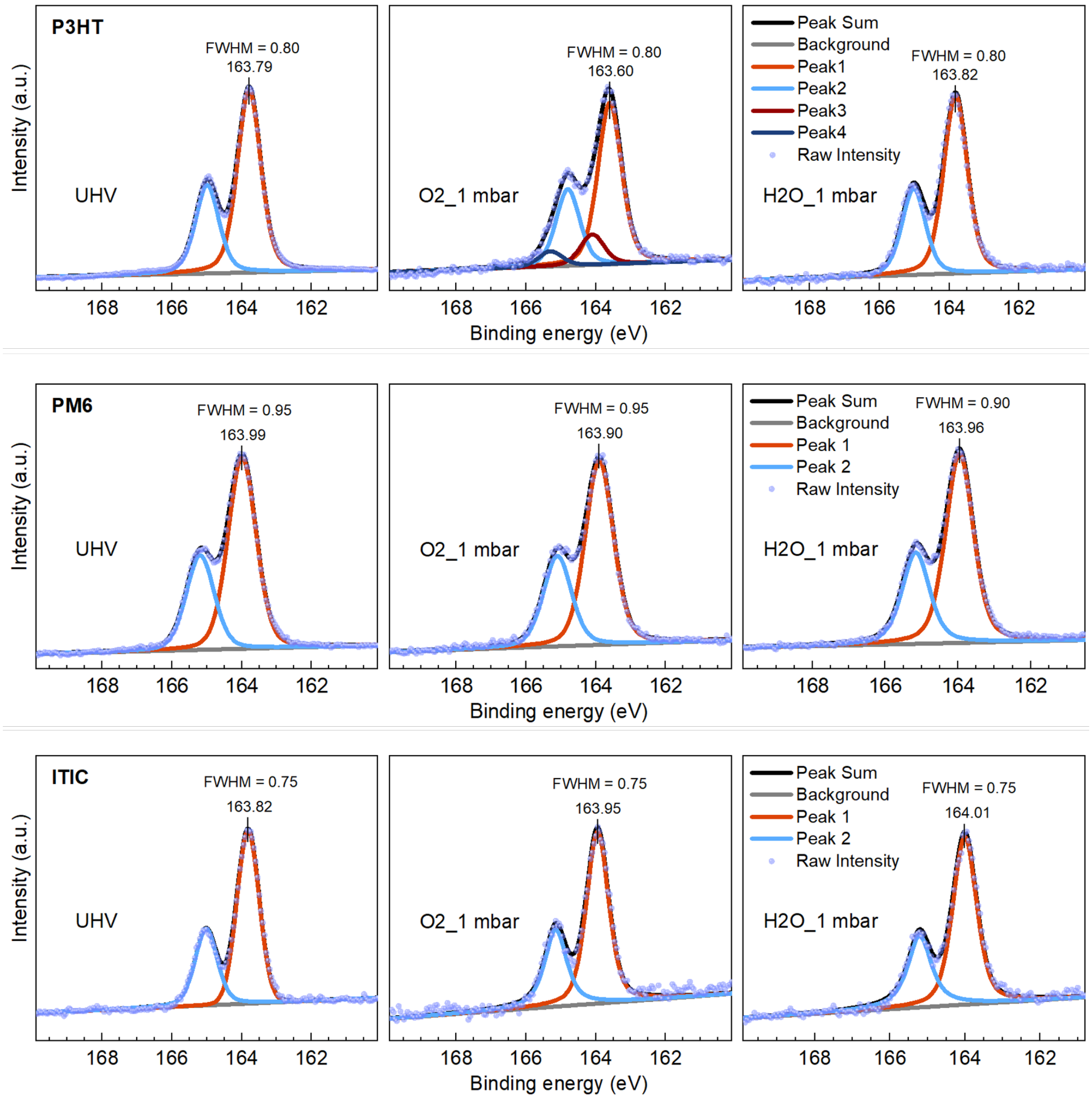

Figure S16. The influence of O_2 and H_2O vapor on the S2p peak of P3HT, PM6, ITIC film, respectively. Films are sequentially exposed to varying atmospheric conditions: UHV → 1 mbar O_2 → HV → 1 mbar H_2O → HV.

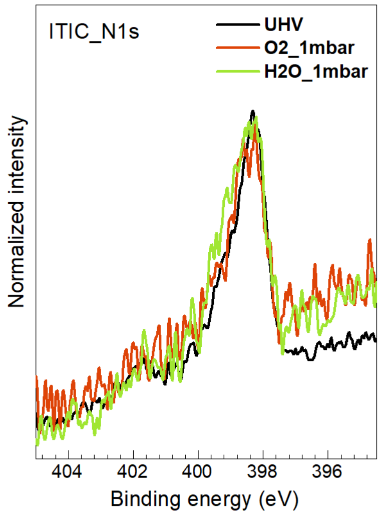

Figure S17. Normalized N1s peaks for ITIC under sequential atmospheric conditions: UHV → 1 mbar O_2 → HV → 1 mbar H_2O → HV. Peaks are aligned to the same binding energy for improved comparison.

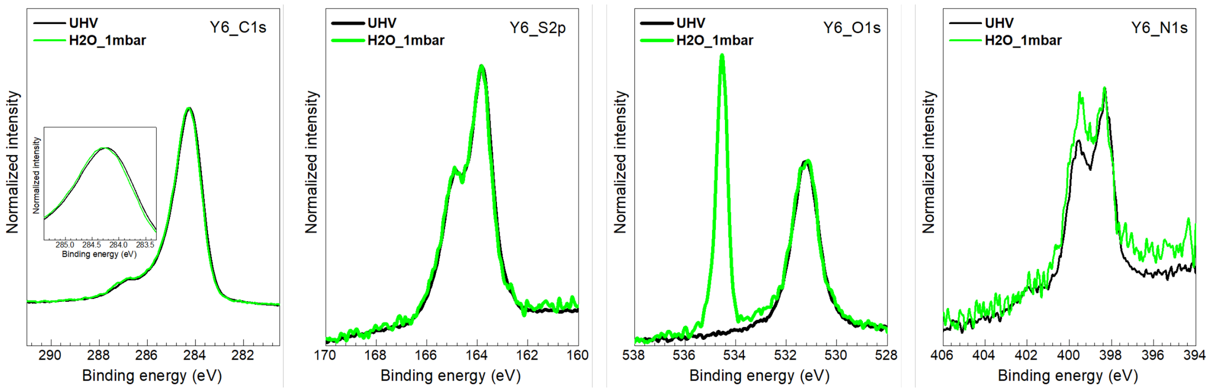

Figure S18. Normalized C1s, S2p, O1s, and N1s peaks for the Y6 film under UHV conditions and after exposure to 1 mbar H_2O. Y6 film is exposed sequentially to ‘UHV → 1 mbar H_2O → HV’.

Table S4. Summary of peak information of P3HT, PM6, ITIC films under different atmospheric conditions from NAP-XPS spectra. S2p peak information is displayed with only the S2p 3/2 peak shown for clarity. The S2p (3/2, 1/2) doublet was fitted with a fixed spin-orbit splitting of 1.2 eV, an area ratio of 2:1 (S2p 3/2 : S2p 1/2), and a consistent FWHM.

**C1s**	Atmospheric condition	Peak 1		Peak 2		Peak 2 area ratio
		Peak position	FWHM	Peak position	FWHM	= Peak2 / (Peak 1 + Peak 2)
Peak attribution		C=C–C, C=C–S		C–O, C=O, C≡N (ITIC)		
P3HT	UHV	284.67	1.1	287.32	1.87	0.031
	O_2_1mbar	284.41	1.22	286.3	2.08	0.090
	HV after O_2	284.46	1.12	286.28	3.18	0.108
	H_2O_1mbar	284.63	1.08	286.62	2.18	0.026
	HV after H_2O	284.64	1.09	-	-	-
PM6	UHV	284.75	1.1	286.72	1.2	0.049
	O_2_1mbar	284.53	1.14	286.52	1.94	0.099
	HV after O_2	284.59	1.07	286.59	1.26	0.064
	H_2O_1mbar	284.69	1.08	286.63	1.04	0.068
	HV after H_2O	-	-	-	-	-
ITIC	UHV	284.16	1.05	285.64	2.15	0.208
	O_2_1mbar	284.24	1.05	285.62	2.35	0.210
	HV after O_2	-	-	-	-	-
	H_2O_1mbar	284.32	1.05	285.62	2.25	0.178
	HV after H_2O	284.16	1.13	285.62	2.17	0.198

**S2p**	Atmospheric condition	Peak 1		Peak 3	
		Peak position	FWHM	Peak position	FWHM
Peak attribution		C–S S2p 3/2		oxygen-interacting S S2p 3/2	
P3HT	UHV	163.79	0.8	-	-
	O_2_1mbar	163.60	0.8	164.1	0.8
	H_2O_1mbar	163.82	0.8	-	-
PM6	UHV	163.99	0.95	-	-
	O_2_1mbar	163.90	0.95	-	-
	H_2O_1mbar	163.96	0.9	-	-
ITIC	UHV	163.82	0.75	-	-
	O_2_1mbar	163.95	0.75	-	-
	H_2O_1mbar	164.01	0.75	-	-

References

(1) Ishii, H.; Hayashi, N.; Ito, E.; Washizu, Y.; Sugi, K.; Kimura, Y.; Niwano, M.; Ouchi, Y.; Seki, K. Kelvin Probe Study of Band Bending at Organic Semiconductor/Metal Interfaces: Examination of Fermi Level Alignment. *Phys. Status Solidi Appl. Res.* **2004**, *201*, 1075–1094.

(2) Ito, E.; Oji, H.; Hayashi, N.; Ishii, H.; Ouchi, Y.; Seki, K. Electronic Structures of TPD/Metal Interfaces Studied by Photoemission and Kelvin Probe Method. *Appl. Surf.*

Sci. **2001**, *175–176*, 407–411. https://doi.org/10.1016/S0169-4332(01)00088-5.
(3) Tal, O.; Rosenwaks, Y.; Preezant, Y.; Tessler, N.; Chan, C. K.; Kahn, A. Direct Determination of the Hole Density of States in Undoped and Doped Amorphous Organic Films with High Lateral Resolution. *Phys. Rev. Lett.* **2005**, *95, 256405*.
(4) Szemjonov, A.; Galkowski, K.; Anaya, M.; Andaji-Garmaroudi, Z.; Baikie, T. K.; Mackowski, S.; Baikie, I. D.; Stranks, S. D.; Islam, M. S. Impact of Oxygen on the Electronic Structure of Triple-Cation Halide Perovskites. *ACS Mater. Lett.* **2019**, *1*, 506–510.
(5) Marquardt Niels. Introduction to the Principles of Vacuum Physics. In *CAS - CERN Accelerator School : Vacuum Technolog*; Turner, S., Ed.; Snekersten, Denmark, 1999; 1–24.
(6) Bertrandie, J.; Han, J.; De Castro, C. S. P.; Yengel, E.; Gorenflot, J.; Anthopoulos, T.; Laquai, F.; Sharma, A.; Baran, D. The Energy Level Conundrum of Organic Semiconductors in Solar Cells. *Adv. Mater.* **2022**, 2202575.
(7) Li, X.; Zhang, Q.; Yu, J.; Xu, Y.; Zhang, R.; Wang, C.; Zhang, H.; Fabiano, S.; Liu, X.; Hou, J.; Gao, F.; Fahlman, M. Mapping the Energy Level Alignment at Donor/Acceptor Interfaces in Non-Fullerene Organic Solar Cells. *Nat. Commun.* **2022**, *13*, 2046.
